# Structural characterization of a Type B chloramphenicol acetyltransferase from the emerging pathogen *Elizabethkingia anophelis* NUHP1

**DOI:** 10.1038/s41598-021-88672-z

**Published:** 2021-05-04

**Authors:** Seyed Mohammad Ghafoori, Alyssa M. Robles, Angelika M. Arada, Paniz Shirmast, David M. Dranow, Stephen J. Mayclin, Donald D. Lorimer, Peter J. Myler, Thomas E. Edwards, Misty L. Kuhn, Jade K. Forwood

**Affiliations:** 1 School of Biomedical Sciences, Charles Sturt University, Wagga Wagga, NSW 2650 Australia; 2 Department of Chemistry and Biochemistry, San Francisco State University, San Francisco, CA USA; 3 Seattle Structural Genomics Center for Infectious Disease, Seattle, WA USA; 4 UCB Pharma, Bainbridge Island, WA USA; 5 Seattle Children’s Research Institute, University of Washington, Seattle, WA USA

**Keywords:** X-ray crystallography, Transferases

## Abstract

*Elizabethkingia anophelis* is an emerging multidrug resistant pathogen that has caused several global outbreaks. *E. anophelis* belongs to the large family of *Flavobacteriaceae,* which contains many bacteria that are plant, bird, fish, and human pathogens. Several antibiotic resistance genes are found within the *E. anophelis* genome*,* including a chloramphenicol acetyltransferase (CAT). CATs play important roles in antibiotic resistance and can be transferred in genetic mobile elements. They catalyse the acetylation of the antibiotic chloramphenicol, thereby reducing its effectiveness as a viable drug for therapy. Here, we determined the high-resolution crystal structure of a CAT protein from the *E. anophelis * NUHP1 strain that caused a Singaporean outbreak. Its structure does not resemble that of the classical Type A CATs but rather exhibits significant similarity to other previously characterized Type B (CatB) proteins from *Pseudomonas aeruginosa, Vibrio cholerae* and *Vibrio vulnificus,* which adopt a hexapeptide repeat fold*.* Moreover, the CAT protein from *E. anophelis* displayed high sequence similarity to other clinically validated chloramphenicol resistance genes, indicating it may also play a role in resistance to this antibiotic. Our work expands the very limited structural and functional coverage of proteins from *Flavobacteriaceae* pathogens which are becoming increasingly more problematic.

## Introduction

*Flavobacteriaceae* is a large family of Gram-negative, mostly aerobic bacteria found in a wide variety of environments^1^. Within the family, some genera contain species that are known to be pathogenic toward plants, fish, birds and humans^2^. For example, the genus *Flavobacterium* has three species, *F. psychrophilum, F. columnare* and *F. branchiophilum*, that cause disease within fish, and one species, *F. johnsoniae,* that infects plants^2,3^. *Tenacibaculum maritimum* also infects fish by causing tenacibaculosis^4^. In ducks, geese and turkeys, *Riemerella anatipestifer* causes serositis and septicaemia^5^. Additionally, *Ornithobacterium rhinotracheale*, *Coenonia anatina,* and *Elizabethkingia meningoseptica* cause respiratory diseases within birds^6–8^*.* Human pathogens from *Flavobacteriaceae* include *Capnocytophaga canimorsus* and *Elizabethkingia spp.* The former is found within the saliva of dogs and cats and causes sepsis, gangrene, meningitis, endocarditis and eye infections; it is transmitted to humans primarily through bites^9^. Currently, the transmission of *Elizabethkingia* pathogens remains unclear, but the bacterium resides in water, in soil, on hospital surfaces, hospital water service lines, and in human patients.

*Elizabethkingia* is an opportunistic, emerging pathogen that has caused recent outbreaks among the general population and immunocompromised patients in Asia and North America, specifically in Singapore, Hong Kong, Taiwan, and the United States (Wisconsin, Illinois and Michigan)^10,11^. It was first isolated in 1949 from infants with septicemia or meningitis and was initially classified under the genus *Flavobacterium*^12^. Later it was reclassified into the genus *Chryseobacterium,* and in 2005 it was given its own genus *Elizabethkingia*^13^. Currently, the genus comprises three aerobic, non-motile rod-shaped Gram-negative species, including *E. miricola, E. meningoseptica,* and *E. anophelis*. Recent studies have identified potential new species that could be added to this genus, but further investigation is required^14^. Multiple *Elizabethkingia* strains have been isolated from a variety of environments, including human patients and mosquitoes^11^. Interestingly, *E. miricola* was isolated in 2003 from condensed water samples obtained from the space station Mir^15^. It has since been reported to cause pneumonia and lower respiratory tract infections, but the mechanism of transmission of the bacterium is unknown^16^. *E. meningoseptica*, previously identified as *Chryseobacterium meningoseptica*, is a hospital acquired pathogen that causes neonatal meningitis, pneumonia, and endocarditis^17,18^. *E. anophelis* is a relatively newly identified bacterium, and some investigations have suggested that it originated from the midgut of the *Anopheles* mosquito, *Anopheles gambiae*^19,20^; however, it has also been isolated from *Anopheles stephensi*^21^. It causes similar infections as *E. meningoseptica,* which has made it challenging to clinically differentiate between these two organisms. As a result, *E. anophelis* infections have been underestimated due to their misclassification^22^.

The *E. anophelis* bacterium responsible for the Singaporean outbreak was isolated from a human patient and designated as the NUHP1 strain. NUHP1 is differentiated from other *E. anophelis* strains because it acquired an ICE*E*a1 integrative conjugative element (ICE) within its *mutY* gene. ICE is a mobile genetic element that integrates into the host chromosome, replicates, excises, and forms a plasmid in order to be transferred to other bacterial cells via horizontal conjugation. These mobile genetic elements are used by bacteria to enhance survival in diverse environments and often contain antibiotic resistance genes. Within the *Flavobacteriaceae* family, only *E. anophelis* and *R. anatipestifer* have been shown to contain ICE^23,24^. Notably, only strains of *E. anophelis* with ICE have caused outbreaks. While ICE contain antibiotic resistance genes, additional antibiotic resistance genes are found outside these regions on the chromosome and contribute to bacterial survival. Within the *E. anophelis* NUHP1 genome, 14 antibiotic resistance genes have been identified, including ones required for resistance to aminoglycosides, beta-lactams, macrolides, tetracycline, trimethoprim, and chloramphenicol (Cm)^25^. One example of an antibiotic resistance gene that is conserved in all *Elizabethkingia* strains is the chloramphenicol acetyltransferase (CAT) gene^26^. To our knowledge this gene has not been described as being located in an ICE.

**Figure 1:**
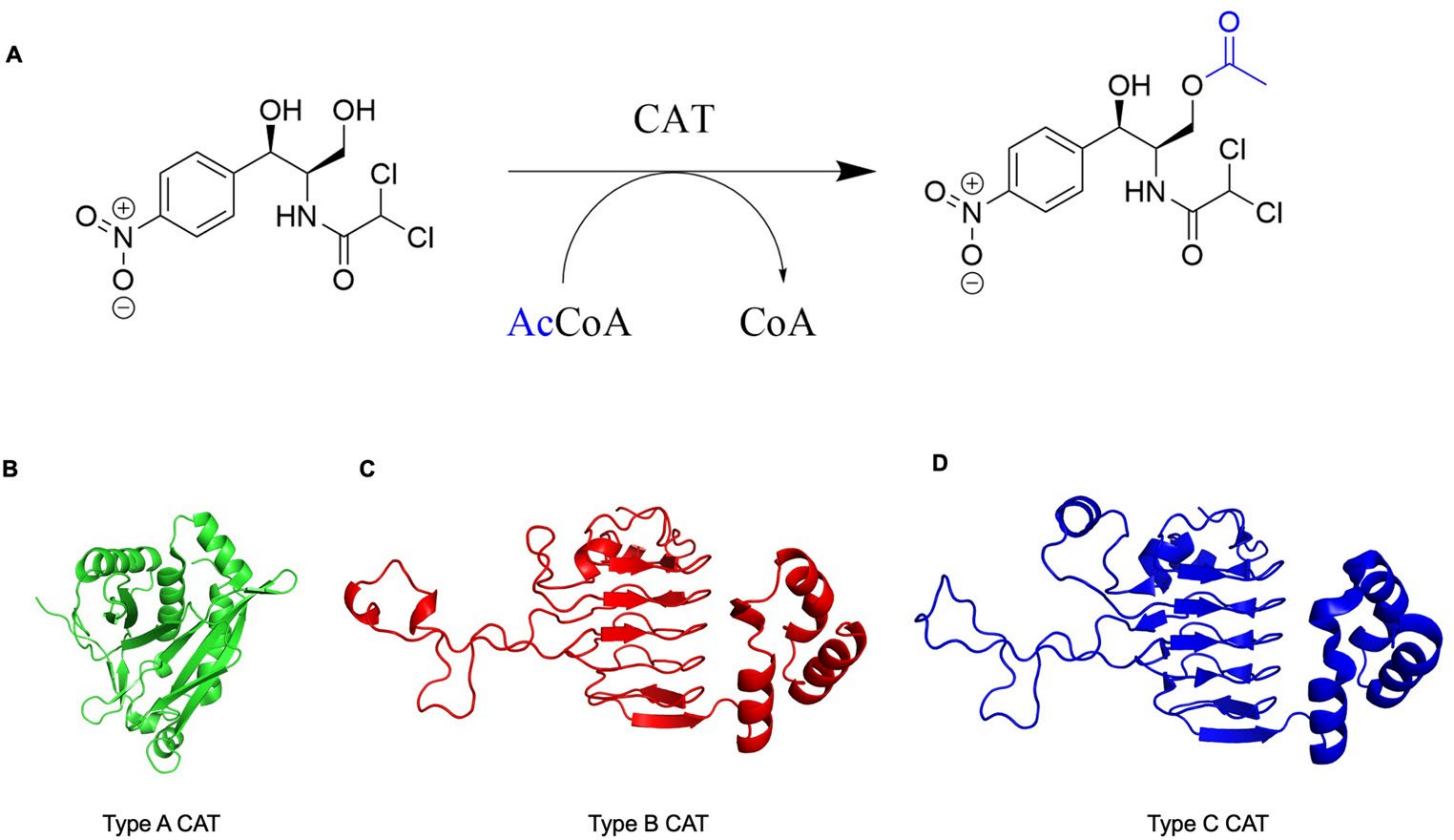
Chloramphenicol acetyltransferase (CAT) reaction and representative types of CAT structures. (**A**) CATs catalyse the *O*-acetylation transfer reaction of an acetyl group from acetyl coenzyme A (AcCoA) to the 3′-position of chloramphenicol. Representative single monomer structures of different types of CAT proteins are also shown. (**B**) Type A CAT from *Escherichia coli* (PDB ID: 3U9F^28^) in green. C) Type B CAT from *Pseudomonas aeruginosa* (PDB ID: 2XAT^29^) in red. D) Type C CAT from *Allivibrio fischeri* (PDB ID: 5UX9^27^) in blue.

Since the *E. anophelis* NUHP1 strain has caused significant outbreaks in recent years, the Seattle Center for Structural Genomics of Infectious Diseases selected antibiotic resistance proteins from this pathogen for structural determination. One of these proteins is CatB, which is predicted to be a chloramphenicol acetyltransferase. Homologs of this protein are important for bacterial Cm antibiotic resistance because they acetylate the 3′-hydroxyl group of Cm (Fig. 1A). This covalent chemical modification prevents Cm from inhibiting bacterial protein synthesis in ribosomes. CAT enzymes are classified based on their origin and their sequence and structural homology. They have been categorized into three predominant types: Type A, Type B and Type C^27^. These are also sometimes referred to as CatA, CatB, and CatC. A defining characteristic between these CATs is that Type B and C display a lower apparent affinity for Cm than Type A^27^. Additionally, Type A CATs form a distinct structural group compared to Type B and C. All three types of proteins are trimers, but Type B and C adopt a hexapeptide repeat structural fold, which is not found in Type A (Fig. 1B–D). Since the *E. anophelis catB* gene is conserved across all *Elizabethkingia* strains and CatB proteins are critical for Cm resistance in important human bacterial pathogens, we explored the structural characteristics of this protein in more detail. This study provides further insight into antibiotic resistance proteins from this important emerging pathogen and adds to the fundamental structural knowledge of CatB proteins.

## Materials and methods

The *E. anophelis* NUHP1 *catB* gene (UniProt ID: A0A077EJ45) was cloned, expressed, and purified as described^30^. In preparation for crystallography, *E. anophelis* CAT was concentrated to 21 mg/ml in 25 mM HEPES/NaOH, pH = 7.0, 500 mM NaCl, 5% glycerol, 2 mM DTT, 0.025% NaN_3_ (SSGCID batch ID ElanA.01572.a.B1.PW38419). The protein was further diluted to 19.5 mg/ml upon the addition of 5 mM MgCl_2_ and 2.5 mM acetyl-CoA and 2.5 mM chloramphenicol and incubated for 10 min at 287 K. Crystals were then grown at 287 K by sitting drop vapor diffusion in XJR trays. A volume of 0.4 μl of protein/ligand complex was mixed with 0.4 μl of JCSG + , well A11 reservoir solution (Rigaku Reagents, Bainbridge Island, WA): 50% (v/v) MPD, 0.1 M Tris base/HCl, pH = 8.5, 0.2 M ammonium phosphate monobasic. The reservoir volume was 80 μl. Crystals were harvested and flash‐frozen in liquid nitrogen. Data were collected at 100 K on a Rayonix MX‐300 mm CCD detector at a wavelength of 0.97872 Å on beamline 21‐ID‐F at Life Sciences Collaborative Access Team (LS‐CAT) at the Advanced Photon Source (APS, Argonne, IL). Data were reduced with the XDS/XSCALE package^31^. The structure was solved using molecular replacement with MorDA^32^ and PDB ID: 1XAT^29^ as a starting model. Iterative rounds of manual model building and automated refinement were carried out using Coot^33^ and Phenix^34^. The quality of the structure was checked by Molprobity^35^, and the final structure was deposited into the Protein Data Bank (PDB) using the code 6MFK. However, the protein was produced with a non-cleavable N-terminal polyhistidine tag (MAHHHHHH) and two C-terminal histidine residues of the tag were partially ordered. The backbone atoms of these His residues and residues 1 and 2 of the protein were ordered. Both the backbone and the side chains of the rest of the residues (3-208) were ordered and included in the model.

## Results

### Structural analysis of *E. anophelis* NUHP1 CAT protein

**Figure 2:**
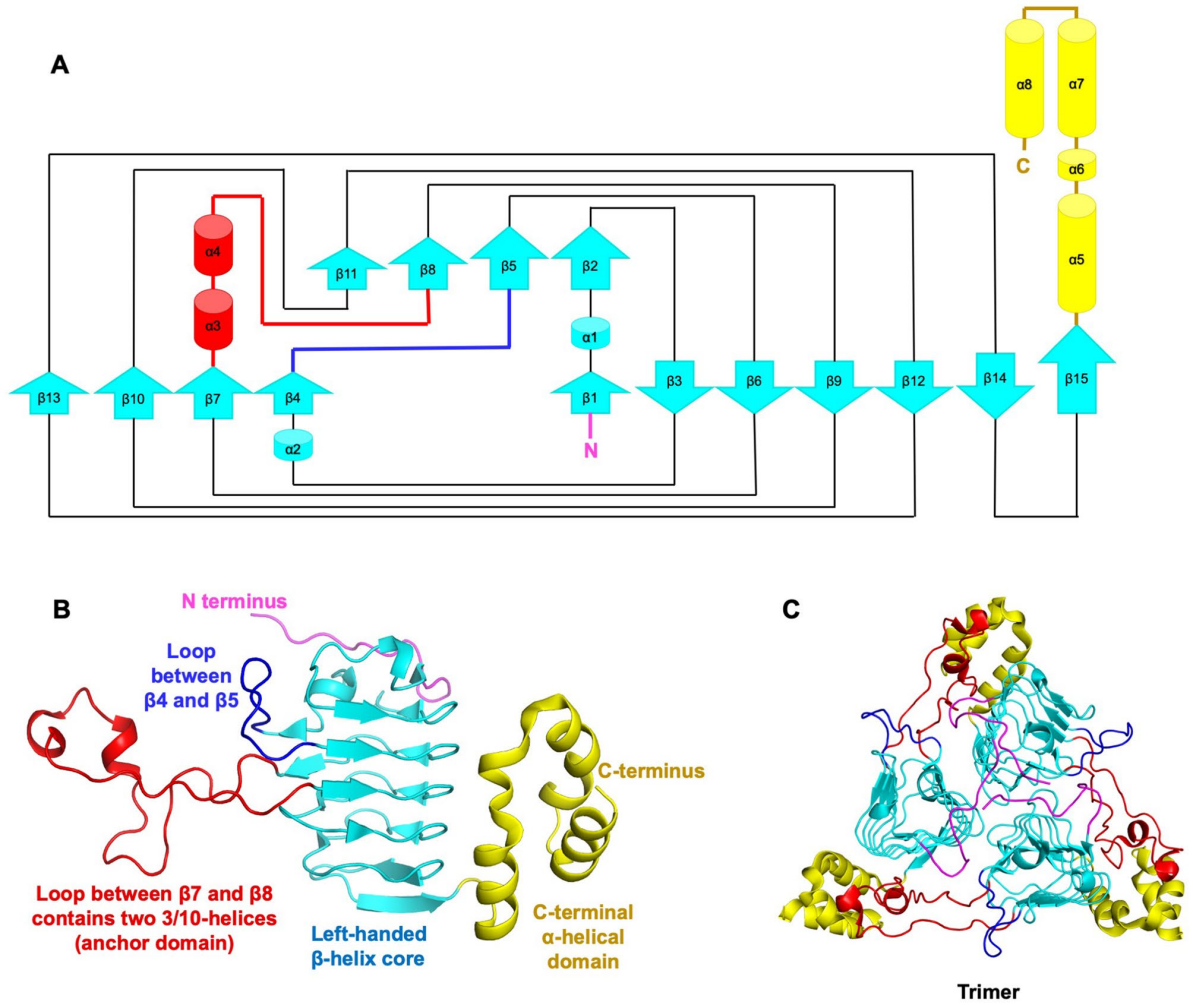
*Elizabethkingia anophelis* chloramphenicol acetyltransferase (CatB) protein structure. (**A**) Topology map. Cyan arrows indicate beta strands, red cylinders indicate 3/10 alpha helices, yellow cylinders represent alpha helices, and lines between shapes represent loops. The N-terminus is shown in pink and the C-terminus is in yellow. (**B**) Tertiary structure of a single monomer. Each domain is coloured as in (**A**). (**C**) Trimeric quaternary structure.

To characterize the type of CAT *E. anophelis* NUHP1 harbors, we determined the structure of the protein encoded by the *catB* gene using X-ray crystallography (Table 1). The electron density map allowed all residues of the protein (1-208) to be modeled. A single protein monomer was present in the asymmetric unit of the crystal with the topology shown in Fig. 2A. The monomer contained two main domains: (1) a left-handed β-helix core (15 β-strands spanning residues 12-163) forms a prism at the center of the monomer and contains two extended loop regions (44-56 and 72-110), and (2) a C-terminal α-helical domain comprised of three α-helices (spanning residues 164-208) (Fig. 2B). To determine whether the *E. anophelis* CAT was likely to form a multimer, we examined the crystal structure by expanding the asymmetric unit and investigated whether large interfaces may be present using Proteins, Interfaces, Structures and Assemblies (PISA)^24^. The only biological assembly predicted from PISA for the *E. anophelis* CAT enzyme was a trimer, which is identical to all other structurally characterized CAT enzymes. (Fig. 2C).

**Table 1:** Data collection and refinement statistics.

Data collection and processing	CatB
Wavelength (Å)	0.97872
Resolution range (Å)	38.4–1.65 (1.69–1.65)
Space group	H32
Unit cell (Å, °)	102.95, 102.95, 115.27, 90, 90, 120
Unique reflections	28,379
Multiplicity	7.882
Completeness (%)	97.5
Mean I/sigma(I)	21.49 (3.9)
Wilson B-factor Å^2^	31.595
R-merge %	4.7 (51.8)
Refinement	
Number of reflections	27,679
Number of R-free reflections	1670
R-work %	14.53
R-free %	16.35
RMS (bonds)	0.007
RMS (angles)	0.832
Ramachandran plot	
Favored (%)	98.1
Allowed (%)	1.9
Outliers (%)	0
PDB accession code	6MFK

To characterize the interfacial residues that are important for this biological assembly, and to reveal the conservation of these residues in other CAT enzymes, we compared these interfaces with other structures of CATs . In our *E. anophelis* CAT structure, we found one interface mediating the trimer formation (Supplementary Figure 1). This interface is mediated through a large number of polar and non-polar interactions. In total, the interface forms 21 hydrogen bonds, two salt bridges (Supplementary Tables 1 and 2), and buries 1699.8 Å^2^ of solvent accessible surface area (ASA). Key binding sites include (1) binding residues within the N-terminal tail A:Met1/B:Met1 (A represents one monomer, B represents another monomer within the biological assembly); (2) seven interactions within the beta-sheet prism; A:Tyr35/B:Ala44,Tyr46, A:Ser32/B:Tyr46, A:Lys162/B:Tyr81, A:Arg140/B:Glu122,Asn156, A:Asn156/B:Asn156, A:Glu122/B:Arg45, A:Asp116/B:Ser85, Ser86,Phe87; and (3) three interactions within the α-helix C-terminal domain (yellow) A:Arg164/B:Tyr81,Tpr83,Ile84, A:Leu193/B:Ile84,Ser85, A:Ser195/B:Ile84. We also identified a large number of hydrophobic and non-bonded contacts at the interface (listed in Supplementary Table 3). Next, we compared this biological interface with related CAT enzymes containing acetyl-CoA (PDB ID: 6U9C^27^), and Cm (PDB ID: 2XAT^29^). We found very similar interfaces in these ligand and cofactor bound structures, with CAT bound to acetyl-CoA exhibiting 23 hydrogen bonds, 2 salt bridges, and 1612 Å^2^ ASA, and CAT bound to Cm exhibiting 16 hydrogen bonds, 2 salt bridges, 1391 Å^2^ ASA (Supplementary Tables 1 and 3).

### The *E. anophelis* protein exhibits greatest structural homology with Type B CATs

To determine which type of CAT the *E. anophelis* protein most closely resembles, we performed a pairwise sequence alignment and a structural comparison with representative sequences and structures of different categories of CATs. To begin, we selected sequences of three different types of CAT proteins that had been structurally characterized: Type A from *E. coli* (catI; UniProt ID: P62577)*,* Type B from *P. aeruginosa* (catB7; UniProt ID: P26841)*,* and Type C from *A. fischeri* (UniProt ID: Q5DZD6)*.* We performed a multiple sequence alignment with the *E. anophelis* CAT and these three proteins and found the *E. anophelis* CAT shared 17%, 62%, and 54% identity with Type A, Type B, and Type C CATs, respectively (Supplementary Figure 2). The insertion typically found in Type C CATs was not present in the *E. anophelis* protein. Therefore, the *E. anophelis* protein most closely resembled Type B CAT protein sequences. To further explore whether the *E. anophelis* protein structure also resembled Type B CATs, we performed a structural comparison of this protein with other types of CATs that had been structurally characterized. We specifically compared the *E. anophelis* 6MFK structure with the *E. coli* 3U9F^28^, *P. aeruginosa* 2XAT^29^, and *A. fischeri* 5UX9^27^ structures. The Type A protein from *E. coli* adopts a completely different fold than the Type B and C proteins from *P. aeruginosa* and *A. fischeri*, respectively; Type B and C CATs adopt a hexapeptide repeat fold (Fig. 1). The *E. anophelis* 6MFK structure superimposed well with the Type B 2XAT structure^29^ (rmsd 0.6 Å) and Type C 5UX9^27^ structure (rmsd 1.1 Å). Therefore, the results of the sequence and structural comparisons indicate the *E. anophelis* protein is most likely a Type B CAT.

### Putative active site residues of the *E. anophelis* CAT protein and other Type B CATs are conserved

**Figure 3:**
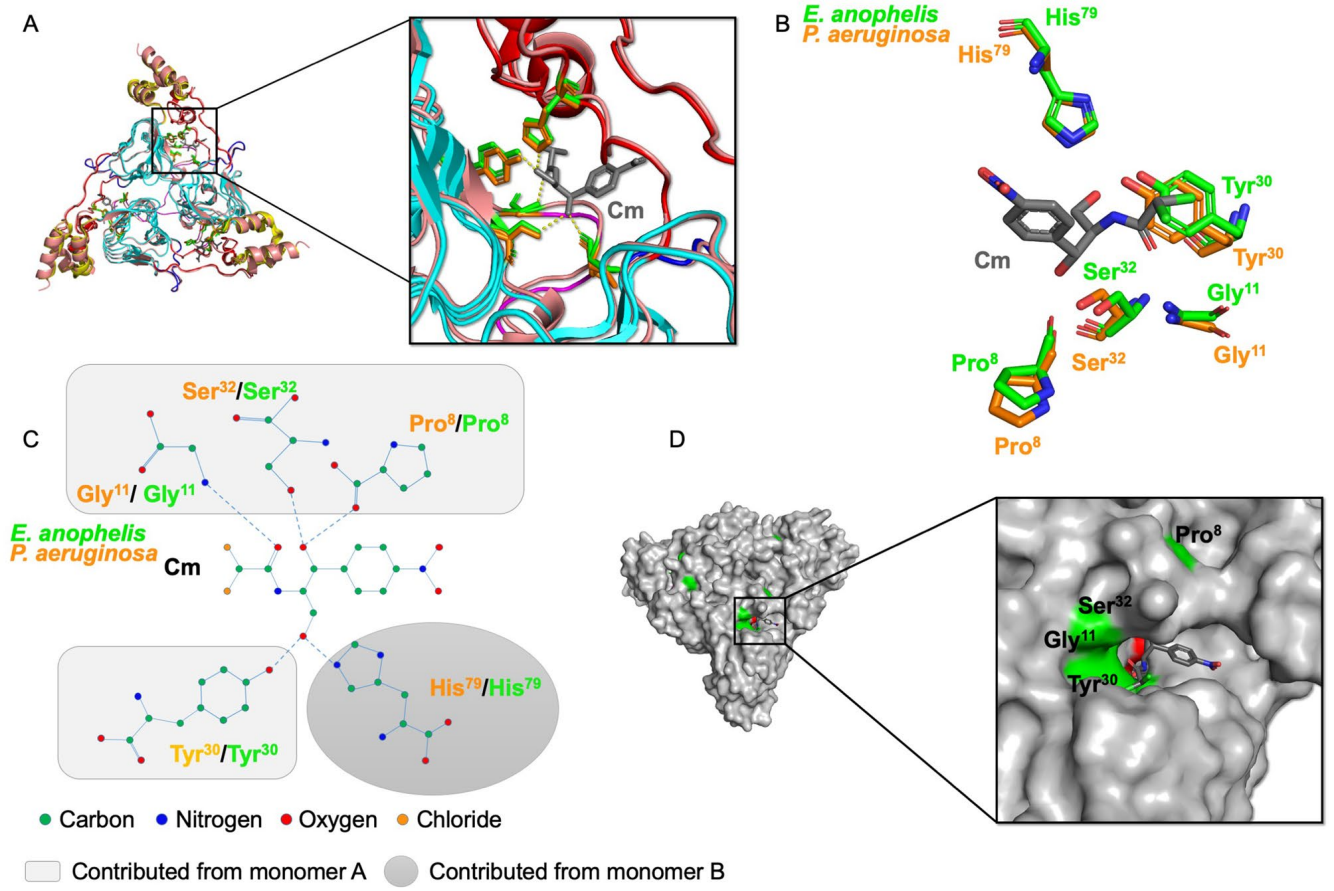
Comparison of the Cm acceptor sites from *E. anophelis* CatB and *P. aeruginosa* catB7. (**A**) Superimposed structure of *E. anophelis* CatB (PDB ID: 6MFK) and *P. aeruginosa* catB7 (PDB ID: 2XAT^29^) trimer. Zoomed view to show H-bonding interactions between putative active site residues and Cm. (**B**) Cm binding site residues of *E. anophelis* and *P. aeruginosa* CAT proteins. Cm is shown in gray sticks. (**C**) Diagram of hydrogen bonds between residues of the *E. anophelis* and *P. aeruginosa* CAT proteins and Cm molecule. (**D**) *E. anopheles* CatB putative Cm acceptor site cavity. Cm is in gray sticks and residues important for H-bonding are coloured in green. Cm was modeled from the *P. aeruginosa* 2XAT^29^ structure.

Next, we examined the putative active site residues of the *E. anophelis* CAT protein and compared them to previously determined Type B CAT structures in complex with substrates or substrate analogs. CAT proteins have two binding sites for each substrate (Cm and AcCoA), which are located at the interface between monomers of the trimer. To determine which residues are located in both sites of the *E. anophelis* CAT, we superimposed its structure with the *P. aeruginosa* catB7 (PDB ID: 2XAT^29^; rmsd 0.6 Å) structure in complex with Cm and desulfo-coenzyme A and the *V. cholerae* catB9 (PDB ID: 6U9C^27^; rmsd 0.6 Å) structure in complex with AcCoA. The trimeric *E. anophelis* CAT structure was built based on the 6U9C^27^ structure and ligands from the 2XAT^29^ and 6U9C^27^ structures were modeled into the trimer to compare putative active site residues. The residues that could potentially hydrogen bond with Cm in the binding site of the *E. anophelis* structure include Pro8, Gly11, Tyr30, and Ser32 from one monomer and His79 from a second monomer (Fig. 3). These residues are all identical in the CAT enzyme from *P. aeruginosa* (PDB ID: 2XAT^29^) (Fig. 3).

**Figure 4:**
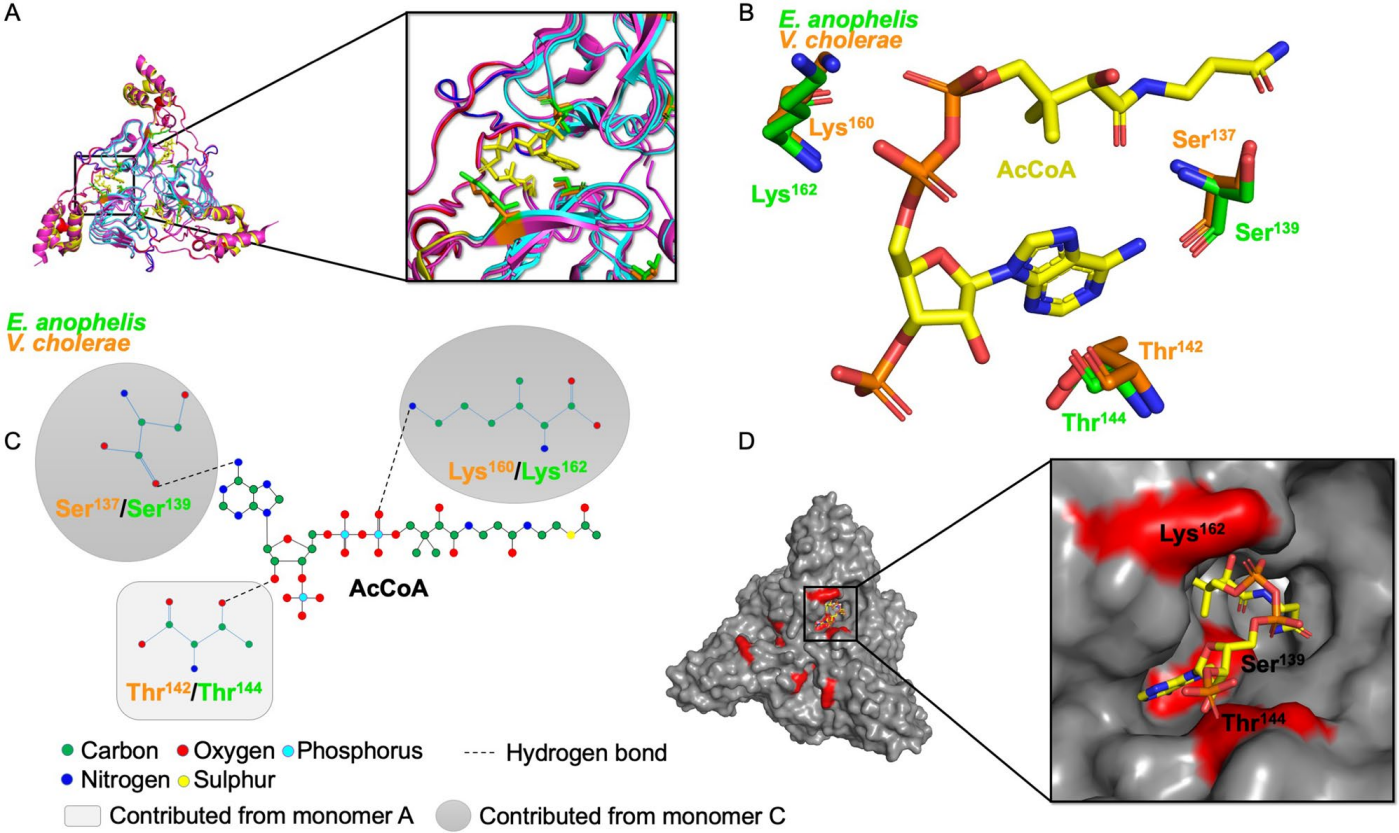
Comparison of the AcCoA donor sites of *E. anophelis* CatB and *V. cholerae* catB7 proteins. (**A**) Superimposed structure of *E. anophelis* CatB (6MFK) and *V. cholerae* catB9 (6U9C^27^) trimer. Zoomed view to show residues surrounding AcCoA, shown in yellow sticks. (**B**) Putative active site residues of *E. anophelis* and *V. cholerae* CAT proteins found within the AcCoA binding site. (**C**) Diagram of hydrogen bonds between putative active site residues of the *E. anophelis* and *V. cholerae* CAT proteins and AcCoA molecule. (**D**) CatB putative active site cavity where AcCoA binds; residues important for H-bonding are coloured in red. AcCoA is in yellow sticks and is modeled from the *V. cholerae* 6U9C^27^ structure.

Residues critical for mediating H-bonding interactions with AcCoA in the 6U9C^27^ structure included Ser137 and Lys160 from the first monomer and Thr142 in the second monomer. The corresponding residues in the 6MFK structure are Ser139, Lys162, Thr144 (Fig. 4).

**Figure 5:**
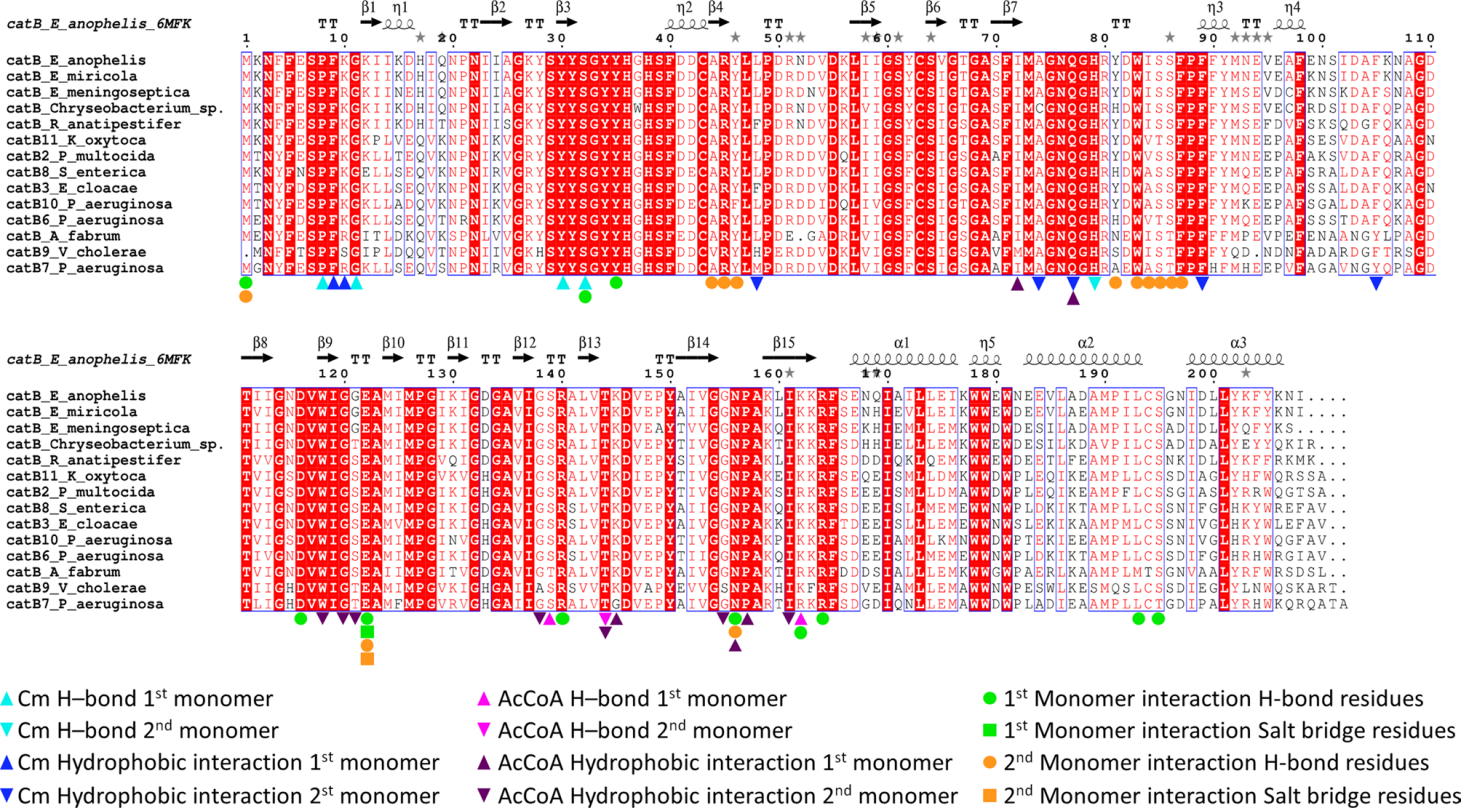
Multiple sequence alignment of *E. anophelis* NUHP1 CAT with various Type B CATs from *Flavobacteriaceae* and validated Cm resistance proteins. The secondary structural elements of the *E. anophelis* NUHP1 protein (PDB ID: 6MFK) are shown above the multiple sequence alignment. Conserved residues are highlighted in red. Residues of the Cm binding site are denoted with cyan (H-bonds) and blue (hydrophobic interactions) symbols, where residues from one monomer are indicated with triangles and residues of the second monomer are shown as inverted triangles. Residues of the AcCoA binding site are denoted with magenta (H-bonds) and purple (hydrophobic) symbols, where residues from one monomer are indicated with triangles and residues of the second monomer are shown as inverted triangles. Green and orange symbols indicate interfacial residues that form H-bonds or salt bridges between protomers. Protein sequences within the alignment include: *Elizabethkingia anophelis* catB (UniProt ID A0A077EJ45), *Elizabethkingia miricola* catB (NCBI Accession KGO08276)*, Elizabethkingia meningoseptica* catB (NCBI Accession QDZ61149)*, Chryseobacterium sp.* catB (UniProt ID A0A3DMIM7), *Riemerella anatipestifer* catB (UniProt ID E5D2K4), *Klebsiella oxytoca KONIH1* catB11 (NCBI Accession AID93387), *Pasterurella multocida* catB2 (UniProt ID Q83ZX9), *Salmonella enterica subsp. enterica serovar Typhi* catB8 (UniProt ID Q79PD0), *Enterobacter cloacae* catB3 (UniProt ID C1IUN4), *Pseudomonas aeruginosa* catB10 (UniProt ID A2Q6I9), *Pseudomonas aeruginosa* catB6 (UniProt ID Q9R818), *Agrobacterium fabrum str. C58* catB (UniProt ID P23364), *Vibrio cholerae* catB9 (UniProt ID H9L3X9), and *Pseudomonas aeruginosa PAO1* catB7 (UniProt ID P26841). The multiple sequence alignment was generated with ESpript (http://espript.ibcp.fr/ESPript/ESPript/).

Once we identified the key putative active site residues in the *E. anophelis* CAT structure, we examined the conservation of these residues across sequences of other Flavobacteriaceae pathogens (Fig. 5). To begin, we aligned the *E. anophelis* CAT protein sequence to others identified by BLASTp against the genomes of the following pathogens: *E. miricola*, *E. meningoseptica*, *Chryseobacterium sp.,* and *R. anatipestifer.* We found the *E. anophelis* CAT protein shares 95% sequence identity with *E. miricola*, 86% identity with *E. meningoseptica*, 83% identity with *Chryseobacterium sp.,* and 81% identity with *R. anatipestifer* Type B CAT proteins. Additionally, the putative active site residues for both Cm and AcCoA sites identified in the *E. anophelis* CAT protein were highly conserved across all of the CAT sequences from these pathogens.

### *E. anophelis* CatB is similar to clinically validated Cm resistance Proteins

To determine whether the *E. anophelis* CAT protein was similar to known Cm resistance proteins in different pathogens, we searched the Comprehensive Antibiotic Resistance Database (CARD)^36^ using the *E. anophelis* CAT protein sequence. The search yielded nine proteins from a variety of bacterial pathogens with validated Cm resistance profiles (Table 2; Fig. 5). All of these proteins were Type B CATs and they shared between 62–73% sequence identity with the *E. anophelis* CAT. When we compared their active site residues with the *E. anophelis* CAT protein, we saw all Cm binding site residues were conserved. The only variation in residue conservation was in the AcCoA binding site. The majority of the residue substitutions were of similar chemical properties (e.g. substituting Val for Ile). However, one exception to this pattern was observed: the CatB7 protein from *P. aeruginosa* had a Gly residue in the corresponding location of Lys145 in the *E. anophelis* protein. Based on these results, it is highly likely that the *E. anophelis* CAT and its homologs in other *Flavobacteriaceae* pathogens could also catalyze the acetylation of Cm and confer resistance to this antibiotic.

**Table 2:** Results of Comprehensive Antibiotic Resistance Database (CARD) query using the *E. anophelis NUHP1* CatB protein as search sequence.

Score	Accession #	Name	E value	% Sequence Identity	Species
307	3004660	catB11	3.67E−107	73	*Klebsiella oxytoca* KONIH1
304	3002675	catB2	8.48E−106	72	*Pasteurella multocida*
300	3002680	catB8	2.60E−104	71	*Salmonella enterica subsp. enterica serovar Typhi*
299	3002676	catB3	6.01E−104	72	*Enterobacter cloacae*
298	3003110	catB10	2.13E−103	70	*Pseudomonas aeruginosa*
295	3002678	catB6	1.80E−102	69	*Pseudomonas aeruginosa*
280	3002681	catB9	2.60E−96	64	*Vibrio cholerae*
278	3004451	*Agrobacterium fabrum* chloramphenicol acetyltransferase	1.25E−95	66	*Agrobacterium fabrum str*. C58
272	3002679	*Pseudomonas aeruginosa* catB7	2.32E−93	63	*Pseudomonas aeruginosa* PAO1

The CARD database can be found at https://card.mcmaster.ca/. Accession numbers correspond to specific proteins in CARD. A total of 23 proteins were identified in the search but only the top nine sequences were annotated as chloramphenicol acetyltransferases and are shown in the table.

## Discussion

*E. anophelis* has been discovered in diverse environmental and host associations, including its presence in soil and water and its isolation from the African and Asian malaria vector mosquitoes *Anopheles gambiae* and *Anopheles stephensi*. Currently it is unclear whether the mosquito itself can be a source of *E. anophelis* transmission to humans, or if other mechanisms occur, including potential reservoirs within hospitals such as sink basins and water faucets^37^. In a study examining different *E. anophelis* strains, those isolated from the midgut of a mosquito contained genes that encode a xylose isomerase and xylulose kinase, which human isolates lacked. This suggests that different environments may have specific requirements for sugar metabolism, and clinical and environmental strains of *E. anophelis* differ from one another^19^. Some other *Flavobacteriaceae* pathogens have been shown to be transmitted in the air, on surfaces, in water, and by ingesting contaminated food^38^. They can also be part of the normal flora of the throat of some duck species^39^.

*E. anophelis* harbors a variety of antibiotic resistance genes along with several multidrug efflux pumps, which are speculated to contribute to the shape and stabilization of the microbial community within the gut of the mosquito^40^. In addition to antibiotic resistance genes, multiple virulence factors contribute to *Elizabethkingia* species survival and persistence. For example, *E. meningoseptica* contains various virulence factors, including those that contribute to proteolysis, iron uptake and transport, biofilms, and capsule formation. Bacterial persistence on surfaces, including cellular adhesion and medical devices, is facilitated by these virulence factors via biofilm, capsule and sometimes curli formation. Biofilms have been shown to be critical for *E. meningoseptica* infections and also allow these bacteria to resist surface disinfection^41^.

Cm has anti-biofilm properties and is effective at killing a variety of bacteria and preventing colony growth^42^; however, this has not been investigated for *Elizabethkingia* species. Cm is not typically used to treat internal bacterial infections due to serious side-effects and toxicity, but it is more widely used as a topical treatment or for some ocular infections^43^. Because Cm is not widely prescribed, some current bacterial pathogens are still susceptible to this antibiotic. Therefore, renewed interest in salvaging or repurposing this drug to treat bacterial infections is also being considered as a viable path forward^44^. While it is unlikely this drug would be used to treat an *E. anophelis* infection in the current pharmaceutical climate, it is possible Cm would be considered in the future as our arsenal of effective antibiotics becomes reduced. Moreover, Cm is an effective drug that can efficiently pass the blood–brain barrier^45^, which is important for treating meningitis infections like those associated with *E. anophelis* or *E. meningoseptica*. Thus, knowing the *E. anophelis* NUHP1 bacterium contains a Cm resistance gene may be important for future therapeutic considerations.

Our analysis of the *E. anophelis* CAT protein has shown it adopts a similar structure and retains conserved putative active site residues as clinically validated Type B CAT proteins in other bacterial pathogens. While we recognize *in vitro* enzyme kinetics assays are needed to ensure the *E. anophelis* enzyme can actually acetylate Cm, the near 100% conservation of putative active site residues compared to other Type B CATs that have been kinetically characterized suggests they are functionally similar. Even though Type B CATs acetylate Cm and are important for Cm resistance, their native functions remain elusive. Several other hexapeptide repeat acetyltransferases have been explored and have been shown to acylate different sugars or cell wall polysaccharides^46,47^. However, these characterized enzymes are still divergent in sequence and structure from the Type B CATs. Therefore, further investigation into the native function of the Type B CAT enzymes is warranted, especially since these proteins are widely found in opportunistic and colonizing pathogens. It would be particularly interesting to explore the native function of the CatB protein from *E. anophelis* since it has an extraordinarily flexible biological capacity to live in a wide array of environments and at times can colonize humans and cause disease.

Despite our lack of knowledge of the native function of the *E. anophelis* CAT protein, the gene is conserved in all identified environmental and nosocomial *E. anophelis* strains^48^. Our analysis of the Type B CAT protein sequence across *Elizabethkinigia* species showed most of the protein sequence is conserved; however, there were some key amino acid changes between the proteins from different species. Due to the difficulty in accurately differentiating *Elizabethkingia* species during genome sequencing^22^, this gene may be used as a tool to improve species identification of isolates and propagation of different strains around the world.

## Supplementary Information

Supplementary Information
