# Supplementary material for: Structural characterization of a Type B chloramphenicol acetyltransferase from the emerging pathogen Elizabethkingia anophelis NUHP1
Source: Sci Rep. 2021 May 4;11:9453. doi: 10.1038/s41598-021-88672-z (PMC8096840; doi:10.1038/s41598-021-88672-z)
Supplement: Supplementary file 1 — Supplementary Information [file 41598_2021_88672_MOESM1_ESM.docx]

**Structural characterization of a Type B chloramphenicol acetyltransferase from the emerging pathogen *Elizabethkingia anophelis* NUHP1**

**Seyed Mohammad Ghafoori^1^, Alyssa M. Robles^2^, Angelika M. Arada^2^, Paniz Shirmast^1^, David M. Dranow^3,4^, Stephen J. Mayclin^3,4^, Donald D. Lorimer^3,4^, Peter S. Horanyi^3,4^, Thomas E. Edwards^3,4^, Misty L. Kuhn^2^, Jade K. Forwood^1^***

**Supplementary Table 1: Comparison between three CAT B interfaces from *V. cholerae* (left column), *E. anophelis* (middle column), and *P. aeruginosa* (right column).** Different number of salt bridges and interface area can be seen between these enzymes.

| **PDB ID: 6U9C**  **Number of H-bonds: 23**  **Number of Salt bonds: 2**  **Interface area [Å^2^]: 1612.7** | | | **PDB ID: 6MFK**  **Number of H-bonds: 21**  **Number of Salt bonds: 2**  **Interface area [Å^2^]: 1699.8** | | | **PDB ID: 2XAT**  **Number of H-bonds: 16**  **Number of Salt bonds: 2**  **Interface area [Å^2^]: 1391.0** | | |
| --- | --- | --- | --- | --- | --- | --- | --- | --- |
| **Protomer 1** | **Dist. [Å]** | **Protomer 2** | **Protomer 1** | **Dist. [Å]** | **Protomer 2** | **Protomer 1** | **Dist. [Å]** | **Protomer 2** |
| B:ARG  44[ NH1] | 3.56 | A:THR 119[ OG1] | A:TYR  35[ OH ] | 3.86 | B:ALA  44[ O  ] | A:TYR  34[ OH ] | 3.82 | B:ASP  42[ OD1] |
| B:ARG  44[ NH1] | 2.93 | A:GLU 120[ OE2] | A:SER  32[ OG ] | 3.08 | B:TYR  46[ OH ] | A:TYR  35[ OH ] | 3.76 | B:ALA  44[ O  ] |
| B:ARG  44[ NH2] | 2.84 | A:GLU 120[ OE2] | A:ARG 164[ NE ] | 2.85 | B:TYR  81[ O  ] | A:ARG 164[ NE ] | 2.96 | B:ALA  81[ O  ] |
| B:TYR  45[ N  ] | 2.98 | A:TYR  34[ OH ] | A:LYS 162[ NZ ] | 2.80 | B:TYR  81[ OH ] | A:ARG 164[ NH2] | 2.91 | B:TRP  83[ O  ] |
| B:TYR  45[ OH ] | 3.83 | A:SER  31[ O  ] | A:ARG 164[ NH2] | 2.72 | B:TRP  83[ O  ] | A:TRP 178[ NE1] | 3.88 | B:ALA  84[ O  ] |
| B:ILE  83[ N  ] | 3.12 | A:SER 193[ O  ] | A:ARG 164[ NH2] | 2.92 | B:ILE  84[ O  ] | A:ARG 140[ NH2] | 3.04 | B:GLU 122[ O  ] |
| B:ILE  83[ N  ] | 3.68 | A:LEU 191[ O  ] | A:ARG 140[ NH1] | 2.80 | B:GLU 122[ O  ] | A:ASN 156[ N  ] | 3.22 | B:ASN 156[ O  ] |
| B:SER  84[ OG ] | 2.71 | A:ASP 114[ OD2] | A:ASN 156[ N  ] | 2.89 | B:ASN 156[ O  ] | A:THR 121[ OG1] | 3.43 | B:ARG  45[ NH1] |
| B:THR  85[ N  ] | 3.32 | A:ASP 114[ OD2] | A:MET   1[ O  ] | 2.95 | B:MET   1[ N  ] | A:GLU 122[ OE2] | 2.96 | B:ARG  45[ NH2] |
| B:THR  85[ OG1] | 3.78 | A:ASP 114[ OD2] | A:GLU 122[ OE2] | 2.84 | B:ARG  45[ NH1] | A:TYR  35[ OH ] | 2.82 | B:TYR  46[ N  ] |
| B:PHE  86[ N  ] | 3.47 | A:ASP 114[ OD2] | A:GLU 122[ OE2] | 2.85 | B:ARG  45[ NH2] | A:THR 195[ O  ] | 2.89 | B:ALA  84[ N  ] |
| B:ASN  95[ ND2] | 2.33 | A:GLU 181[ O  ] | A:TYR  35[ OH ] | 2.82 | B:TYR  46[ N  ] | A:LEU 193[ O  ] | 3.79 | B:ALA  84[ N  ] |
| B:ARG 100[ NH2] | 3.48 | A:GLN 189[ O  ] | A:SER  32[ O  ] | 3.46 | B:TYR  46[ OH ] | A:GLU 116[ OE2] | 2.67 | B:THR  86[ N  ] |
| B:ASN 154[ ND2] | 3.27 | A:ARG 138[ O  ] | A:SER 195[ O  ] | 2.82 | B:ILE  84[ N  ] | A:GLU 116[ OE2] | 3.44 | B:THR  86[ OG1] |
| B:VAL  43[ O  ] | 3.65 | A:TYR  34[ OH ] | A:LEU 193[ O  ] | 3.59 | B:ILE  84[ N  ] | A:CYS 194[ SG ] | 3.75 | B:VAL 102[ N  ] |
| B:SER  80[ O  ] | 3.48 | A:ARG 162[ NH2] | A:LEU 193[ O  ] | 3.77 | B:SER  85[ N  ] | A:ARG 140[ O  ] | 3.31 | B:ASN 156[ ND2] |
| B:SER  80[ O  ] | 3.23 | A:ARG 162[ NE ] | A:ASP 116[ OD1] | 2.72 | B:SER  85[ OG ] |  |  |  |
| B:TRP  82[ O  ] | 2.93 | A:ARG 162[ NH2] | A:ASP 116[ OD1] | 2.96 | B:SER  86[ N  ] |  |  |  |
| B:ILE  83[ O  ] | 3.41 | A:ARG 162[ NH2] | A:ASP 116[ OD1] | 3.35 | B:SER  86[ OG ] |  |  |  |
| B:ASN  95[ OD1] | 3.08 | A:LYS 185[ N  ] | A:ASP 116[ OD1] | 3.78 | B:PHE  87[ N  ] |  |  |  |
| B:GLU 120[ O  ] | 2.90 | A:ARG 138[ NH1] | A:ARG 140[ O  ] | 2.94 | B:ASN 156[ ND2] |  |  |  |
| B:GLU 120[ O  ] | 3.14 | A:ARG 138[ NH2] |  |  |  |  |  |  |
| B:ASN 154[ O  ] | 2.45 | A:SER 153[ OG ] |  |  |  |  |  |  |
| **Salt Bridges** | | | **Salt Bridges** | | | **Salt Bridges** | | |
| *B:ARG 44[ NH1]* | 2.93 | *A:GLU 120[ OE2]* | *A:GLU 122[ OE2]* | 2.84 | *A:ARG 45[ NH1]* | *A:GLU 122[ OE2]* | 3.43 | *A:ARG 45[ NH1]* |
| *B:ARG 44[ NH2]* | 2.84 | *A:GLU 120[ OE2]* | *A:GLU 122[ OE2]* | 2.85 | *A:ARG 45[ NH2]* | *A:GLU 122[ OE2]* | 2.96 | *A:ARG 45[ NH2]* |

**Supplementary Table 2**: Interaction between different domains between monomers. Residues are coloured based on domain colouring in Figure 2.

| **Hydrogen bonds** | | | |
| --- | --- | --- | --- |
| **##** | **Monomer A** | **Dist. [Å]** | **Monomer B** |
| **1** | **A:TYR  35[ OH ]** | **3.86** | **B:ALA  44[ O  ]** |
| **2** | **A:SER  32[ OG ]** | **3.08** | **B:TYR  46[ OH ]** |
| **3** | **A:ARG 164[ NE ]** | **2.85** | **B:TYR  81[ O  ]** |
| **4** | **A:LYS 162[ NZ ]** | **2.80** | **B:TYR  81[ OH ]** |
| **5** | **A:ARG 164[ NH2]** | **2.72** | **B:TRP  83[ O  ]** |
| **6** | **A:ARG 164[ NH2]** | **2.92** | **B:ILE  84[ O  ]** |
| **7** | **A:ARG 140[ NH1]** | **2.80** | **B:GLU 122[ O  ]** |
| **8** | **A:ASN 156[ N  ]** | **2.89** | **B:ASN 156[ O  ]** |
| **9** | **A:MET   1[ O  ]** | **2.95** | **B:MET   1[ N  ]** |
| **10** | **A:GLU 122[ OE2]** | **2.84** | **B:ARG  45[ NH1]** |
| **11** | **A:GLU 122[ OE2]** | **2.85** | **B:ARG  45[ NH2]** |
| **12** | **A:TYR  35[ OH ]** | **2.82** | **B:TYR  46[ N  ]** |
| **13** | **A:SER  32[ O  ]** | **3.46** | **B:TYR  46[ OH ]** |
| **14** | **A:LEU 193[ O  ]** | **3.59** | **B:ILE  84[ N  ]** |
| **15** | **A:SER 195[ O  ]** | **2.82** | **B:ILE  84[ N  ]** |
| **16** | **A:LEU 193[ O  ]** | **3.77** | **B:SER  85[ N  ]** |
| **17** | **A:ASP 116[ OD1]** | **2.72** | **B:SER  85[ OG ]** |
| **18** | **A:ASP 116[ OD1]** | **2.96** | **B:SER  86[ N  ]** |
| **19** | **A:ASP 116[ OD1]** | **3.34** | **B:SER  86[ OG ]** |
| **20** | **A:ASP 116[ OD1]** | **3.78** | **B:PHE  87[ N  ]** |
| **21** | **A:ARG 140[ O  ]** | **2.94** | **B:ASN 156[ ND2]** |
| **Salt bridges** | | | |
| **1** | **A:GLU 122[ OE2]** | **2.84** | **B:ARG  45[ NH1]** |
| **2** | **A:GLU 122[ OE2]** | **2.85** | **B:ARG  45[ NH2]** |

**Supplementary Table 3: Comparison between non-bonded contacts of three CAT B interfaces from *V. cholerae* (left column), *E. anophelis* (middle column), and *P. aeruginosa* (right column).**

| PDB ID: 6U9C  Number of non-bonded contacts: 208 | | | PDB ID: 6MFK  Number of non-bonded contacts: 228 | | | PDB ID: 2XAT  Number of non-bonded contacts: 161 | | |
| --- | --- | --- | --- | --- | --- | --- | --- | --- |
| Protomer 1 | **Dist. [Å]** | **Protomer 2** | **Protomer 1** | **Dist. [Å]** | **Protomer 2** | **Protomer 1** | **Dist. [Å]** | **Protomer 2** |
| A:PRO 7[O] | 3.53 | B:TYR 45[CD1] | A:MET 1[O] | 2.95 | B:MET 1[N] | A:PRO 8[O] | 3.86 | B:TYR 46[CD1] |
| A:PRO 7[O] | 3.22 | B:TYR 45[CE1] | A:MET 1[O] | 3.78 | B:MET 1[CA] | A:PRO 8[O] | 3.86 | B:TYR 46[CE1] |
| A:PHE 8[CD1] | 3.88 | B:HIS 47[NE2] | A:MET 1[O] | 3.51 | B:MET 1[CB] | A:PHE 9[CE1] | 3.50 | B:TYR 46[CA] |
| A:PHE 8[CD2] | 3.78 | B:HIS 47[CE1] | A:PRO 8[O] | 3.39 | B:TYR 46[CD1] | A:PHE 9[CE1] | 3.36 | B:TYR 46[C] |
| A:PHE 8[CE1] | 3.72 | B:TYR 45[CA] | A:PRO 8[O] | 3.02 | B:TYR 46[CE1] | A:PHE 9[CE1] | 3.03 | B:TYR 46[O] |
| A:PHE 8[CE1] | 3.71 | B:TYR 45[C] | A:PHE 9[CE1] | 3.60 | B:TYR 46[CA] | A:PHE 9[CE1] | 3.86 | B:TYR 46[CB] |
| A:PHE 8[CE1] | 3.38 | B:TYR 45[O] | A:PHE 9[CE1] | 3.59 | B:TYR 46[C] | A:PHE 9[CE1] | 3.62 | B:MET 48[CG] |
| A:PHE 8[CE1] | 3.86 | B:TYR 45[CB] | A:PHE 9[CE1] | 3.35 | B:TYR 46[O] | A:PHE 9[CZ] | 3.64 | B:TYR 46[CA] |
| A:PHE 8[CE1] | 3.79 | B:HIS 47[CD2] | A:PHE 9[CE1] | 3.89 | B:LEU 48[CG] | A:PHE 9[CZ] | 3.31 | B:TYR 46[C] |
| A:PHE 8[CE1] | 3.57 | B:HIS 47[CE1] | A:PHE 9[CE1] | 3.61 | B:LEU 48[CD2] | A:PHE 9[CZ] | 3.28 | B:TYR 46[O] |
| A:PHE 8[CE1] | 3.29 | B:HIS 47[NE2] | A:PHE 9[CE2] | 3.70 | B:LEU 48[CD2] | A:PHE 9[CZ] | 3.81 | B:LEU 47[N] |
| A:PHE 8[CE2] | 3.54 | B:HIS 47[ND1] | A:PHE 9[CZ] | 3.87 | B:TYR 46[CA] | A:PHE 9[CZ] | 3.75 | B:LEU 47[C] |
| A:PHE 8[CE2] | 3.48 | B:HIS 47[CE1] | A:PHE 9[CZ] | 3.68 | B:TYR 46[C] | A:PHE 9[CZ] | 3.85 | B:LEU 47[O] |
| A:PHE 8[CZ] | 3.81 | B:TYR 45[CA] | A:PHE 9[CZ] | 3.71 | B:TYR 46[O] | A:PHE 9[CZ] | 3.84 | B:MET 48[N] |
| A:PHE 8[CZ] | 3.56 | B:TYR 45[C] | A:PHE 9[CZ] | 3.84 | B:LEU 48[N] | A:PHE 9[CZ] | 3.34 | B:MET 48[CG] |
| A:PHE 8[CZ] | 3.46 | B:TYR 45[O] | A:PHE 9[CZ] | 3.87 | B:LEU 48[CA] | A:TYR 28[O] | 3.44 | B:PHE87[CE1] |
| A:PHE 8[CZ] | 3.88 | B:HIS 47[N] | A:PHE 9[CZ] | 3.89 | B:LEU 48[CG] | A:TYR 28[O] | 3.51 | B:PHE 87[CZ] |
| A:PHE 8[CZ] | 3.65 | B:HIS 47[CG] | A:PHE 9[CZ] | 3.50 | B:LEU 48[CD2] | A:TYR 28[CD1] | 3.33 | B:MET 92[CE] |
| A:PHE 8[CZ] | 3.48 | B:HIS 47[ND1] | A:TYR 28[O] | 3.54 | B:PHE 87[CE1] | A:TYR 28[CD1] | 3.61 | B:PHE 98[CE2] |
| A:PHE 8[CZ] | 3.58 | B:HIS 47[CD2] | A:TYR 28[O] | 3.38 | B:PHE 87[CZ] | A:TYR 28[CD1] | 3.83 | B:PHE 98[CZ] |
| A:PHE 8[CZ] | 3.32 | B:HIS 47[CE1] | A:TYR 28[CB] | 3.78 | B:PHE 87[CZ] | A:TYR 28[CE1] | 3.86 | B:MET 92[CE] |
| A:PHE 8[CZ] | 3.37 | B:HIS 47[NE2] | A:TYR 28[CD1] | 3.51 | B:MET 92[CE] | A:TYR 28[CE1] | 3.58 | B:PHE 98[CE2] |
| A:HIS 27[O] | 3.22 | B:PHE 86[CE1] | A:TYR 28[CD1] | 3.85 | B:PHE 98[CZ] | A:TYR 28[CE1] | 3.61 | B:PHE 98[CZ] |
| A:HIS 27[O] | 3.22 | B:PHE 86[CZ] | A:TYR 28[CE1] | 3.89 | B:VAL 95[CG1] | A:TYR 28[CZ] | 3.82 | B:PHE 98[CZ] |
| A:HIS 27[ND1] | 3.87 | B:ASN 93[OD1] | A:TYR 28[CE1] | 3.49 | B:VAL 95[CG2] | A:TYR 30[OH] | 3.46 | B:HIS 79[CE1] |
| A:HIS 27[CD2] | 3.76 | B:PHE 96[CZ] | A:TYR 28[CE1] | 3.88 | B:PHE 98[CZ] | A:TYR 30[OH] | 3.78 | B:HIS 79[NE2] |
| A:HIS 27[CE1] | 3.49 | B:ASN 93[OD1] | A:TYR 28[OH] | 3.58 | B:VAL 95[CG1] | A:TYR 30[OH] | 3.34 | B:PRO 88[CG] |
| A:HIS 27[NE2] | 3.70 | B:PHE 96[CZ] | A:TYR 30[CD1] | 3.71 | B:PHE 78[CE1] | A:TYR 30[OH] | 3.90 | B:PRO 88[CD] |
| A:TYR 29[CD1] | 3.66 | B:PHE 86[CE1] | A:TYR 30[CE1] | 3.83 | B:PHE 78[CE1] | A:TYR 34[OH] | 3.82 | B:ASP 42[OD1] |
| A:TYR 29[CD1] | 3.81 | B:GLN 91[NE2] | A:TYR 30[CE1] | 3.82 | B:PRO 88[CG] | A:TYR 35[CG] | 3.61 | B:ARG 45[CD] |
| A:TYR 29[CE1] | 3.71 | B:PHE 86[CE1] | A:TYR 30[CE1] | 3.89 | B:PRO 88[CD] | A:TYR 35[CD2] | 3.37 | B:ARG 45[CD] |
| A:TYR 29[CE1] | 3.83 | B:GLN 91[NE2] | A:TYR 30[OH] | 3.06 | B:HIS 79[CE1] | A:TYR 35[CD2] | 3.66 | B:TYR 46[CE1] |
| A:TYR 29[OH] | 3.25 | B:HIS 78[CE1] | A:TYR 30[OH] | 3.38 | B:HIS 79[NE2] | A:TYR 35[CE2] | 3.65 | B:ARG 45[CA] |
| A:TYR 29[OH] | 3.51 | B:HIS 78[NE2] | A:TYR 30[OH] | 3.52 | B:PRO 88[CG] | A:TYR 35[CE2] | 3.49 | B:ARG 45[CB] |
| A:TYR 29[OH] | 3.41 | B:PRO 87[CG] | A:SER 32[O] | 3.46 | B:TYR 46[OH] | A:TYR 35[CE2] | 3.72 | B:ARG 45[CD] |
| A:TYR 29[OH] | 3.74 | B:PRO 87[CD] | A:SER 32[OG] | 3.66 | B:TYR 46[CE1] | A:TYR 35[CE2] | 3.52 | B:TYR 46[N] |
| A:SER 31[O] | 3.83 | B:TYR 45[OH] | A:SER 32[OG] | 3.80 | B:TYR 46[CZ] | A:TYR 35[CE2] | 3.49 | B:TYR 46[CD1] |
| A:TYR 34[CG] | 3.84 | B:ARG 44[CG] | A:SER 32[OG] | 3.08 | B:TYR 46[OH] | A:TYR 35[CE2] | 3.36 | B:TYR 46[CE1] |
| A:TYR 34[CG] | 3.84 | B:ARG 44[CD] | A:TYR 34[CE2] | 3.87 | B:ASP 42[OD1] | A:TYR 35[CZ] | 3.60 | B:ARG 45[CA] |
| A:TYR 34[CD1] | 3.70 | B:ASP 41[O] | A:TYR 35[CD1] | 3.65 | B:ASP 42[O] | A:TYR 35[CZ] | 3.57 | B:TYR 46[N] |
| A:TYR 34[CD1] | 3.75 | B:ARG 44[CG] | A:TYR 35[CE1] | 3.53 | B:ASP 42[O] | A:TYR 35[OH] | 3.76 | B:ALA 44[O] |
| A:TYR 34[CD2] | 3.85 | B:ARG 44[CG] | A:TYR 35[CE2] | 3.83 | B:ARG 45[CB] | A:TYR 35[OH] | 3.38 | B:ARG 45[CA] |
| A:TYR 34[CE1] | 3.62 | B:ASP 41[O] | A:TYR 35[CE2] | 3.88 | B:TYR 46[N] | A:TYR 35[OH] | 3.56 | B:ARG 45[C] |
| A:TYR 34[CE1] | 3.69 | B:ARG 44[CG] | A:TYR 35[CE2] | 3.78 | B:TYR 46[CE2] | A:TYR 35[OH] | 2.82 | B:TYR 46[N] |
| A:TYR 34[CE2] | 3.87 | B:ARG 44[CB] | A:TYR 35[CE2] | 3.72 | B:TYR 46[CZ] | A:TYR 35[OH] | 3.82 | B:TYR 46[CA] |
| A:TYR 34[CE2] | 3.80 | B:ARG 44[CG] | A:TYR 35[CZ] | 3.60 | B:ARG 45[CA] | A:PHE 62[CA] | 3.64 | B:PHE 87[CD1] |
| A:TYR 34[CE2] | 3.89 | B:TYR 45[N] | A:TYR 35[CZ] | 3.64 | B:TYR 46[N] | A:PHE 62[CA] | 3.74 | B:PHE 87[CE1] |
| A:TYR 34[CE2] | 3.81 | B:TYR 45[CE1] | A:TYR 35[OH] | 3.86 | B:ALA 44[O] | A:PHE 62[C] | 3.55 | B:PHE 87[CD1] |
| A:TYR 34[CZ] | 3.62 | B:ARG 44[CA] | A:TYR 35[OH] | 3.41 | B:ARG 45[CA] | A:PHE 62[C] | 3.87 | B:PHE 87[CE1] |
| A:TYR 34[CZ] | 3.88 | B:ARG 44[CB] | A:TYR 35[OH] | 3.60 | B:ARG 45[C] | A:PHE 62[O] | 3.17 | B:PHE 87[CD1] |
| A:TYR 34[CZ] | 3.72 | B:ARG 44[CG] | A:TYR 35[OH] | 2.82 | B:TYR 46[N] | A:PHE 62[O] | 3.90 | B:PHE 87[CE1] |
| A:TYR 34[CZ] | 3.74 | B:TYR 45[N] | A:TYR 35[OH] | 3.75 | B:TYR 46[CA] | A:PHE 62[CB] | 3.77 | B:PHE 87[CD1] |
| A:TYR 34[OH] | 3.65 | B:VAL 43[O] | A:HIS 36[CE1] | 3.87 | B:ARG 45[NH2] | A:PHE 62[CE1] | 3.81 | B:PHE 89[CE1] |
| A:TYR 34[OH] | 3.38 | B:ARG 44[CA] | A:TYR 62[CA] | 3.55 | B:PHE 87[CD1] | A:SER 64[CB] | 3.65 | B:HIS 79[CE1] |
| A:TYR 34[OH] | 3.63 | B:ARG 44[C] | A:TYR 62[CA] | 3.82 | B:PHE 87[CE1] | A:SER 64[OG] | 3.77 | B:HIS 79[CE1] |
| A:TYR 34[OH] | 2.98 | B:TYR 45[N] | A:TYR 62[C] | 3.34 | B:PHE 87[CD1] | A:SER 64[OG] | 3.68 | B:PRO 88[CD] |
| A:PHE 61[CA] | 3.74 | B:PHE 86[CD1] | A:TYR 62[C] | 3.84 | B:PHE 87[CE1] | A:GLU 116[CG] | 3.75 | B:SER 85[OG] |
| A:PHE 61[C] | 3.56 | B:PHE 86[CD1] | A:TYR 62[O] | 3.78 | B:PHE 87[CB] | A:GLU 116[CD] | 3.89 | B:ALA 84[O] |
| A:PHE 61[O] | 3.64 | B:PHE 86[CB] | A:TYR 62[O] | 3.88 | B:PHE 87[CG] | A:GLU 116[CD] | 3.62 | B:SER 85[CA] |
| A:PHE 61[O] | 3.82 | B:PHE 86[CG] | A:TYR 62[O] | 3.30 | B:PHE 87[CD1] | A:GLU 116[CD] | 3.84 | B:SER 85[OG] |
| A:PHE 61[O] | 3.50 | B:PHE 86[CD1] | A:TYR 62[CD1] | 3.73 | B:PHE 89[CE1] | A:GLU 116[CD] | 3.70 | B:THR 86[N] |
| A:PHE 61[CD1] | 3.74 | B:PHE 86[CD2] | A:TYR 62[CE1] | 3.87 | B:PHE 89[CE1] | A:GLU 116[OE1] | 3.58 | B:ALA 84[O] |
| A:PHE 61[CD1] | 3.82 | B:PHE 86[CE2] | A:CYS 63[O] | 3.88 | B:PHE 87[CD1] | A:GLU 116[OE2] | 3.63 | B:ALA 84[O] |
| A:CYS 62[O] | 3.81 | B:PHE 86[CE1] | A:CYS 63[O] | 3.54 | B:PHE 87[CE1] | A:GLU 116[OE2] | 2.97 | B:SER 85[CA] |
| A:SER 63[CB] | 3.47 | B:HIS 78[CE1] | A:SER 64[CB] | 3.45 | B:HIS 79[CE1] | A:GLU 116[OE2] | 3.29 | B:SER 85[C] |
| A:SER 63[OG] | 3.52 | B:HIS 78[CE1] | A:SER 64[OG] | 3.64 | B:HIS 79[ND1] | A:GLU 116[OE2] | 3.84 | B:SER 85[CB] |
| A:SER 63[OG] | 3.86 | B:PHE 86[CA] | A:SER 64[OG] | 3.26 | B:HIS 79[CE1] | A:GLU 116[OE2] | 3.77 | B:SER 85[OG] |
| A:SER 63[OG] | 3.74 | B:PHE 86[CD1] | A:SER 64[OG] | 3.77 | B:PHE 87[CD1] | A:GLU 116[OE2] | 2.67 | B:THR 86[N] |
| A:SER 63[OG] | 3.71 | B:PRO 87[CD] | A:SER 64[OG] | 3.56 | B:PRO 88[CD] | A:GLU 116[OE2] | 3.80 | B:THR 86[CA] |
| A:SER 66[GB] | 3.76 | B:TYR 45[OH] | A:THR 67[CB] | 3.80 | B:TYR 46[OH] | A:GLU 116[OE2] | 3.85 | B:THR 86[CB] |
| A:ASP 114[CG] | 3.78 | B:SER 84[CA] | A:THR 67[CG2] | 3.82 | B:ARG 45[NH1] | A:GLU 116[OE2] | 3.44 | B:THR 86[OG1] |
| A:ASP 114[CG] | 3.73 | B:SER 84[CB] | A:ASP 116[CG] | 3.82 | B:SER 85[CA] | A:GLU 116[OE2] | 3.70 | B:THR 86[CG2] |
| A:ASP 114[CG] | 3.47 | B:SER 84[OG] | A:ASP 116[CG] | 3.55 | B:SER 85[OG] | A:TRP 118[CB] | 3.79 | B:THR 86[OG1] |
| A:ASP 114[OD1] | 3.57 | B:SER 84[CA] | A:ASP 116[CG] | 3.83 | B:SER 86[N] | A:TRP 118[NE1] | 3.85 | B:HIS 79[CG] |
| A:ASP 114[OD1] | 3.82 | B:SER 84[C] | A:ASP 116[OD1] | 3.42 | B:SER 85[CA] | A:TRP 118[NE1] | 3.83 | B:HIS 79[CD2] |
| A:ASP 114[OD1] | 3.39 | B:SER 84[CB] | A:ASP 116[OD1] | 3.67 | B:SER 85[C] | A:TRP 118[NE1] | 3.87 | B:HIS 79[NE2] |
| A:ASP 114[OD1] | 2.71 | B:SER 84[OG] | A:ASP 116[OD1] | 3.50 | B:SER 85[CB] | A:TRP 118[CE2] | 3.73 | B:HIS 79[CB] |
| A:ASP 114[OD1] | 3.32 | B:THR 85[N] | A:ASP 116[OD1] | 2.72 | B:SER 85[OG] | A:TRP 118[CE2] | 3.54 | B:HIS 79[CG] |
| A:ASP 114[OD1] | 3.78 | B:THR 85[OG1] | A:ASP 116[OD1] | 2.96 | B:SER 86[N] | A:TRP 118[CE2] | 3.79 | B:HIS 79[CD2] |
| A:ASP 114[OD1] | 3.47 | B:PHE 86[N] | A:ASP 116[OD1] | 3.35 | B:SER 86[OG] | A:TRP 118[CZ2] | 3.41 | B:HIS 79[CB] |
| A:ASP 114[OD1] | 3.84 | B:PHE 86[CB] | A:ASP 116[OD1] | 3.78 | B:PHE 87[N] | A:TRP 118[CZ2] | 3.51 | B:HIS 79[CG] |
| A:ASP 114[OD2] | 3.52 | B:ILE 83[O] | A:ASP 116[OD2] | 3.44 | B:ILE 84[O] | A:TRP 118[CZ2] | 3.62 | B:HIS 79[CD2] |
| A:ASP 114[OD2] | 3.26 | B:SER 84[CA] | A:ASP 116[OD2] | 3.59 | B:SER 85[CA] | A:TRP 118[CZ3] | 3.68 | B:HIS 79[CB] |
| A:ASP 114[OD2] | 3.60 | B:SER 84[CB] | A:ASP 116[OD2] | 3.88 | B:SER 86[N] | A:TRP 118[CH2] | 3.36 | B:HIS 79[CB] |
| A:ASP 114[OD2] | 3.78 | B:SER 84[OG] | A:TRP 118[CB] | 3.48 | B:SER 86[OG] | A:THR 121[OG1] | 3.43 | B:ARG 45[NH1] |
| A:ASP 114[OD2] | 3.77 | B:THR 85[N] | A:TRP 118[CD2] | 3.90 | B:HIS 79[CB] | A:THR 121[CG2] | 3.88 | B:MET 124[CE] |
| A:TRP 116[CB] | 3.43 | B:THR 85[OG1] | A:TRP 118[NE1] | 3.81 | B:HIS 79[CG] | A:GLU 122[OE2] | 3.64 | B:ARG 45[CZ] |
| A:TRP 116[NE1] | 3.81 | B:HIS 78[CD2] | A:TRP 118[NE1] | 3.62 | B:HIS 79[CD2] | A:GLU 122[OE2] | 3.43 | B:ARG 45[NH1] |
| A:TRP 116[CE2] | 3.72 | B:HIS 78[CG] | A:TRP 118[NE1] | 3.74 | B:HIS 79[NE2] | A:GLU 122[OE2] | 2.96 | B:ARG 45[NH2] |
| A:TRP 116[CE2] | 3.67 | B:HIS 78[CD2] | A:TRP 118[CE2] | 3.75 | B:HIS 79[CB] | A:ILE 136[CD1] | 3.37 | B:ALA 81[CB] |
| A:TRP 116[CZ2] | 3.64 | B:HIS 78[CB] | A:TRP 118[CE2] | 3.57 | B:HIS 79[CG] | A:SER 139[O] | 3.72 | B:LEU 142[CD2] |
| A:TRP 116[CZ2] | 3.69 | B:HIS 78[CG] | A:TRP 118[CE2] | 3.65 | B:HIS 79[CD2] | A:SER 139[OG] | 3.84 | B:MET 126[SD] |
| A:TRP 116[CZ2] | 3.53 | B:HIS 78[CD2] | A:TRP 118[CE3] | 3.89 | B:HIS 79[CB] | A:ARG 140[O] | 3.31 | B:ASN 156[ND2] |
| A:TRP 116[CZ3] | 3.67 | B:HIS 78[CB] | A:TRP 118[CZ2] | 3.83 | B:ASN 76[O] | A:ARG 140[CB] | 3.83 | B:LEU 142[CD1] |
| A:TRP 116[CH2] | 3.47 | B:HIS 78[CB] | A:TRP 118[CZ2] | 3.60 | B:HIS 79[CB] | A:ARG 140[CB] | 3.73 | B:ASN 156[ND2] |
| A:THR 119[OG1] | 3.56 | B:ARG 44[NH1] | A:TRP 118[CZ2] | 3.70 | B:HIS 79[CG] | A:ARG 140[CG] | 3.61 | B:MET 124[SD] |
| A:THR 119[CG2] | 3.90 | B:MET 71[CE] | A:TRP 118[CZ2] | 3.63 | B:HIS 79[CD2] | A:ARG 140[CG] | 3.87 | B:LEU 142[CD1] |
| A:GLU 120[CD] | 3.83 | B:ARG 44[NH1] | A:TRP 118[CZ3] | 3.72 | B:HIS 79[CB] | A:ARG 140[CZ] | 3.71 | B:GLU 122[O] |
| A:GLU 120[OE2] | 3.31 | B:ARG 44[CZ] | A:TRP 118[CH2] | 3.58 | B:HIS 79[CB] | A:ARG 140[NH1] | 3.52 | B:GLU 122[O] |
| A:GLU 120[OE2] | 2.93 | B:ARG 44[NH1] | A:GLU 122[CD] | 3.89 | B:ARG 45[NH1] | A:ARG 140[NH2] | 3.04 | B:GLU 122[O] |
| A:GLU 120[OE2] | 2.84 | B:ARG 44[NH2] | A:GLU 122[OE2] | 3.26 | B:ARG 45[CZ] | A:GLY 155[CA] | 3.60 | B:ASN 156[O] |
| A:ILE 134[CD1] | 3.63 | B:SER 80[CB] | A:GLU 122[OE2] | 2.84 | B:ARG 45[NH1] | A:GLY 155[CA] | 3.52 | B:ASN 156[OD1] |
| A:ILE 134[CD1] | 3.81 | B:THR 85[CG2] | A:GLU 122[OE2] | 2.85 | B:ARG 45[NH2] | A:ASN 156[N] | 3.22 | B:ASN 156[O] |
| A:SER 137[CB] | 3.68 | B:MET 124[CE] | A:GLU 122[OE2] | 3.60 | B:MET 124[CE] | A:ASN 156[N] | 3.89 | B:ASN 156[CB] |
| A:ARG 138[O] | 3.27 | B:ASN 154[ND2] | A:SER 139[CB] | 3.87 | B:MET 124[SD] | A:ASN 156[N] | 3.86 | B:ASN 156[CG] |
| A:ARG 138[CB] | 3.75 | B:VAL 140[CG2] | A:ARG 140[C] | 3.73 | B:ASN 156[ND2] | A:ASN 156[CB] | 3.79 | B:ASN 156[CB] |
| A:ARG 138[CG] | 3.85 | B:MET 122[CE] | A:ARG 140[O] | 2.94 | B:ASN 156[ND2] | A:ARG 164[CG] | 3.65 | B:ALA 81[O] |
| A:ARG 138[CG] | 3.79 | B:VAL 140[CG2] | A:ARG 140[CB] | 3.69 | B:ASN 156[ND2] | A:ARG 164[CD] | 3.79 | B:ALA 81[O] |
| A:ARG 138[NE] | 3.66 | B:MET 122[CE] | A:ARG 140[CZ] | 3.54 | B:GLU 122[O] | A:ARG 164[NE] | 2.96 | B:ALA 81[O] |
| A:ARG 138[CZ] | 3.45 | B:GLU 120[O] | A:ARG 140[CZ] | 3.89 | B:MET 124[CE] | A:ARG 164[CZ] | 3.82 | B:ALA 81[O] |
| A:ARG 138[NH1] | 2.90 | B:GLU 120[O] | A:ARG 140[NH1] | 2.80 | B:GLU 122[O] | A:ARG 164[CZ] | 3.63 | B:THR 86[CG2] |
| A:ARG 138[NH2] | 3.14 | B:GLU 120[O] | A:ARG 140[NH1] | 3.90 | B:GLU 122[CB] | A:ARG 164[NH2] | 3.80 | B:ALA 81[O] |
| A:SER 153[CA] | 3.47 | B:ASN 154[O] | A:ARG 140[NH1] | 3.86 | B:ARG 140[CD] | A:ARG 164[NH2] | 2.91 | B:TRP 83[O] |
| A:SER 153[CA] | 3.87 | B:ASN 154[CG] | A:ARG 140[NH2] | 3.42 | B:GLU 122[O] | A:ARG 164[NH2] | 3.48 | B:ALA 84[C] |
| A:SER 153[CA] | 3.79 | B:ASN 154[OD1] | A:ILE 152[CD1] | 3.85 | B:TYR 81[CD1] | A:ARG 164[NH2] | 3.34 | B:ALA 84[O] |
| A:SER 153[CA] | 3.76 | B:ASN 154[ND2] | A:GLY 155[N] | 3.77 | B:ASN 156[OD1] | A:ARG 164[NH2] | 3.69 | B:SER 85[N] |
| A:SER 153[C] | 3.76 | B:ASN 154[ND2] | A:GLY 155[CA] | 3.36 | B:ASN 156[O] | A:ARG 164[NH2] | 3.83 | B:SER 85[CA] |
| A:SER 153[CB] | 3.15 | B:ASN 154[O] | A:GLY 155[CA] | 3.85 | B:ASN 156[CG] | A:ARG 164[NH2] | 3.88 | B:THR 86[N] |
| A:SER 153[OG] | 3.64 | B:ASN 154[C] | A:GLY 155[CA] | 3.29 | B:ASN 156[OD1] | A:ARG 164[NH2] | 3.64 | B:THR 86[CG2] |
| A:SER 153[OG] | 2.45 | B:ASN 154[O] | A:GLY 155[CA] | 3.90 | B:PRO 157[CD] | A:PHE 165[CE1] | 3.82 | B:GLU 82[O] |
| A:ASN 154[N] | 3.83 | B:ASN 154[ND2] | A:GLY 155[C] | 3.62 | B:ASN 156[O] | A:PHE 165[CE2] | 3.79 | B:TRP 83[O] |
| A:ASN 154[CB] | 3.73 | B:ASN 154[ND2] | A:GLY 155[C] | 3.89 | B:ASN 156[CG] | A:PHE 165[CZ] | 3.58 | B:TRP 83[C] |
| A:ARG 162[NE] | 3.23 | B:SER 80[O] | A:GLY 155[C] | 3.80 | B:ASN 156[OD1] | A:PHE 165[CZ] | 3.04 | B:TRP 83[O] |
| A:ARG 162[CZ] | 3.82 | B:SER 80[O] | A:GLY 155[C] | 3.89 | B:ASN 156[ND2] | A:PHE 165[CZ] | 3.79 | B:ALA 84[CA] |
| A:ARG 162[CZ] | 3.46 | B:THR 85[CG2] | A:ASN 156[N] | 2.89 | B:ASN 156[O] | A:TRP 178[NE1] | 3.88 | B:ALA 84[O] |
| A:ARG 162[NH1] | 3.88 | B:ILE 83[O] | A:ASN 156[N] | 3.72 | B:ASN 156[CG] | A:TRP 178[CZ2] | 3.19 | B:SER 85[CB] |
| A:ARG 162[NH2] | 3.48 | B:SER 80[O] | A:ASN 156[N] | 3.82 | B:ASN 156[ND2] | A:TRP 178[CZ2] | 2.97 | B:SER 85[OG] |
| A:ARG 162[NH2] | 2.93 | B:TRP 82[O] | A:ASN 156[CA] | 3.90 | B:ASN 156[O] | A:TRP 178[CZ3] | 3.71 | B:PHE 89[CZ] |
| A:ARG 162[NH2] | 3.74 | B:ILE 83[C] | A:ASN 156[O] | 3.74 | B:ASN 156[O] | A:TRP 178[CH2] | 3.62 | B:SER 85[CB] |
| A:ARG 162[NH2] | 3.41 | B:ILE 83[O] | A:ASN 156[CB] | 3.65 | B:ASN 156[ND2] | A:TRP 178[CH2] | 3.02 | B:SER 85[OG] |
| A:ARG 162[NH2] | 3.78 | B:SER 84[C] | A:LYS 162[CE] | 3.81 | B:TYR 81[CE1] | A:LEU 183[CG] | 3.50 | B:ALA 97[CB] |
| A:ARG 162[NH2] | 3.53 | B:THR 85[N] | A:LYS 162[CE] | 3.78 | B:TYR 81[CZ] | A:LEU 183[CD1] | 3.84 | B:ALA 97[CB] |
| A:ARG 162[NH2] | 3.23 | B:THR 85[CG2] | A:LYS 162[CE] | 3.28 | B:TYR 81[OH] | A:LEU 183[CD1] | 3.67 | B:PHE 89[CE1] |
| A:PHE 163[CE1] | 3.78 | B:ASP 81[O] | A:LYS 162[NZ] | 3.74 | B:TYR 81[CE1] | A:LEU 183[CD2] | 3.56 | B:ALA 97[CB] |
| A:PHE 163[CE1] | 3.81 | B:TRP 82[O] | A:LYS 162[NZ] | 3.65 | B:TYR 81[CZ] | A:GLU 187[CA] | 3.82 | B:ALA 97[O] |
| A:PHE 163[CZ] | 3.86 | B:TRP 82[C] | A:LYS 162[NZ] | 2.80 | B:TYR 81[OH] | A:GLU 187[CB] | 3.44 | B:ALA 97[O] |
| A:PHE 163[CZ] | 3.08 | B:TRP 82[O] | A:ARG 164[CB] | 3.81 | B:TYR 81[O] | A:GLU 187[CG] | 3.10 | B:ALA 97[O] |
| A:TRP 176[CZ2] | 3.47 | B:SER 84[CB] | A:ARG 164[CG] | 3.76 | B:TYR 81[O] | A:GLU 187[CD] | 3.80 | B:ALA 97[CA] |
| A:TRP 176[CZ2] | 3.72 | B:SER 84[OG] | A:ARG 164[CD] | 3.85 | B:TYR 81[O] | A:GLU 187[CD] | 3.73 | B:ALA 97[O] |
| A:TRP 176[CH2] | 3.84 | B:SER 84[OG] | A:ARG 164[NE] | 3.77 | B:TYR 81[C] | A:GLU 187[OE2] | 3.84 | B:ALA 97[O] |
| A:GLU 181[CA] | 3.50 | B:ASN 95[ND2] | A:ARG 164[NE] | 2.84 | B:TYR 81[O] | A:MET 190[CE] | 3.33 | B:PHE 89[CB] |
| A:GLU 181[C] | 3.00 | B:ASN 95[ND2] | A:ARG 164[CZ] | 3.57 | B:TYR 81[O] | A:MET 190[CE] | 3.41 | B:PHE 89[CG] |
| A:GLU 181[O] | 3.24 | B:ASN 95[CG] | A:ARG 164[CZ] | 3.80 | B:ILE 84[O] | A:MET 190[CE] | 3.33 | B:PHE 89[CD2] |
| A:GLU 181[O] | 3.38 | B:ASN 95[OD1] | A:ARG 64[NH2] | 3.40 | B:TYR 81O[] | A:MET 190[CE] | 3.57 | B:ALA 101[CA] |
| A:GLU 181[O] | 2.33 | B:ASN 95[ND2] | A:ARG 164[NH2] | 3.90 | B:TRP 83[C] | A:MET 190[CE] | 3.38 | B:ALA 101[CB] |
| A:GLU 181[CB] | 3.45 | B:ASN 95[ND2] | A:ARG 164[NH2] | 2.72 | B:TRP 83[O] | A:LEU 193[O] | 3.79 | B:ALA 84[N] |
| A:LEU 184[C] | 3.70 | B:ASN 95[OD1] | A:ARG 164[NH2] | 3.43 | B:ILE 84[C] | A:CYS 194[CA] | 3.71 | B:TRP 83[CE3] |
| A:LEU 184[CB] | 3.53 | B:ASN 95[OD1] | A:ARG 164[NH2] | 2.92 | B:ILE 84[O] | A:CYS 194[C] | 3.70 | B:TRP 83[CD2] |
| A:LYS 185[N] | 3.08 | B:ASN 95[OD1] | A:ARG 164[NH2] | 3.87 | B:SER 85[N] | A:CYS 194[O] | 3.68 | B:TRP 83[CD2] |
| A:LYS 185[CA] | 3.78 | B:ASN 95[O] | A:ARG 164[NH2] | 3.82 | B:SER 85[CA] | A:CYS 194[O] | 3.53 | B:TRP 83[CE2] |
| A:LYS 185[CA] | 3.46 | B:ASN 95[OD1] | A:ARG 164[NH2] | 3.72 | B:SER 85[C] | A:CYS 194[O] | 3.77 | B:TRP 83[CZ2] |
| A:LYS 185[CB] | 3.64 | B:ASN 95[O] | A:ARG 164[NH2] | 3.67 | B:SER 86[N] | A:CYS 194[SG] | 3.75 | B:VAL 102[N] |
| A:LYS 185[CB] | 3.72 | B:ASN 95[OD1] | A:PHE 165[CE1] | 3.68 | B:ASP 82[O] | A:THR 195[C] | 3.83 | B:TRP 83[CA] |
| A:LYS 185[CG] | 3.65 | B:ASN 95[O] | A:PHE 165[CZ] | 3.84 | B:TYR 81[O] | A:THR 195[C] | 3.87 | B:TRP 83[CD1] |
| A:LYS 185[CG] | 3.32 | B:ASN 95[CG] | A:PHE 165[CZ] | 3.87 | B:TRP 83[C] | A:THR 195[O] | 3.18 | B:TRP 83[CA] |
| A:LYS 185[CG] | 3.12 | B:ASN 95[OD1] | A:PHE 165[CZ] | 3.29 | B:TRP 83[O] | A:THR 195[O] | 3.46 | B:TRP 83[C] |
| A:LYS 185[CG] | 3.28 | B:ASN 95[ND2] | A:TRP 178[NE1] | 3.81 | B:ILE 84[CG2] | A:THR 195[O] | 3.58 | B:TRP 83[CB] |
| A:LYS 185[CE] | 3.84 | B:ASN 95[O] | A:TRP 178[CZ2] | 3.63 | B:SER 85[CB] | A:THR 195[O] | 2.89 | B:ALA 84[N] |
| A:MET 188[SD] | 3.81 | B:PHE 88[CG] | A:TRP 178[CZ2] | 3.45 | B:SER 85[OG] | A:GLY 196[CA] | 3.33 | B:GLU 82[O] |
| A:MET 188[SD] | 3.63 | B:PHE 88[CD1] | A:TRP 178[CH2] | 3.77 | B:SER 85[CB] | A:ILE 198[CD1] | 3.58 | B:ALA 84[CB] |
| A:MET 188[CE] | 3.35 | B:PHE 88[CB] | A:TRP 178[CH2] | 3.53 | B:SER 85[OG] | A:LEU 201[CD2] | 3.89 | B:ALA 84[CB] |
| A:MET 188[CE] | 3.52 | B:PHE 88[CG] | A:GLU 183[O] | 3.78 | B:ALA 97[CB] |  |  |  |
| A:MET 188[CE] | 3.34 | B:PHE 88[CD1] | A:LEU 186[CD2] | 3.74 | B:PHE 98[CE1] |  |  |  |
| A:MET 188[CE] | 3.44 | B:PHE 96[CA] | A:ALA 187[CA] | 3.41 | B:ALA 97[O] |  |  |  |
| A:MET 188[CE] | 3.35 | B:PHE 96[CB] | A:ALA 187[CB] | 3.90 | B:ALA 97[CA] |  |  |  |
| A:MET 188[CE] | 3.70 | B:PHE 96[CG] | A:ALA 187[CB] | 3.65 | B:ALA 97[O] |  |  |  |
| A:MET 188[CE] | 3.58 | B:PHE 96[CD1] | A:MET 190[CG] | 3.76 | B:ASN 100[C] |  |  |  |
| A:MET 188[CE] | 3.69 | B:ALA 99[CB] | A:MET 190[CG] | 3.40 | B:SER 101[N] |  |  |  |
| A:GLN 189[O] | 3.48 | B:ARG 100[NH2] | A:MET 190[CG] | 3.55 | B:SER 101[CA] |  |  |  |
| A:GLN 189[CG] | 3.86 | B:ARG 100[NH2] | A:MET 190[CG] | 3.83 | B:SER 101[CB] |  |  |  |
| A:SER 190[O] | 3.89 | B:ILE 83[CD1] | A:MET 190[SD] | 3.71 | B:PHE 89[CG] |  |  |  |
| A:LEU 191[O] | 3.68 | B:ILE 83[N] | A:MET 190[SD] | 3.88 | B:PHE 89[CD2] |  |  |  |
| A:LEU 191[O] | 3.87 | B:ILE 83[CB] | A:MET 190[SD] | 3.90 | B:SER 101[CB] |  |  |  |
| A:CYS192[CA] | 3.61 | B:TRP 82[CE3] | A:MET 190[CE] | 3.66 | B:ALA 97[O] |  |  |  |
| A:CYS192[C] | 3.52 | B:TRP 82[CD2] | A:MET 190[CE] | 3.87 | B:PHE 98[CD1] |  |  |  |
| A:CYS192[C] | 3.74 | B:TRP 82[CE2] | A:ILE 192[O] | 3.43 | B:ILE 84[CD1] |  |  |  |
| A:CYS192[C] | 3.68 | B:TRP 82[CE3] | A:LEU 193[O] | 3.59 | B:ILE 84[N] |  |  |  |
| A:CYS192[O] | 3.59 | B:TRP 82[CD2] | A:LEU 193[O] | 3.74 | B:ILE 84[CB] |  |  |  |
| A:CYS192[O] | 3.37 | B:TRP 82[CE2] | A:LEU 193[O] | 3.77 | B:SER 85[N] |  |  |  |
| A:CYS192[O] | 3.81 | B:TRP 82[CD3] | A:CYS 194[CA] | 3.60 | B:TRP 83[CE3] |  |  |  |
| A:CYS192[O] | 3.41 | B:TRP 82[CZ2] | A:CYS 194[C] | 3.53 | B:TRP 83[CD2] |  |  |  |
| A:CYS192[O] | 3.81 | B:TRP 82[CZ3] | A:CYS 194[C] | 3.85 | B:TRP 83[CE2] |  |  |  |
| A:CYS192[O] | 3.62 | B:TRP 82[CH2] | A:CYS 194[C] | 3.58 | B:TRP 83[CE3] |  |  |  |
| A:CYS192[O] | 3.85 | B:ARG 100[CD] | A:CYS 194[O] | 3.65 | B:TRP 83[CD2] |  |  |  |
| A:CYS192[O] | 3.41 | B:ARG 100[NE] | A:CYS 194[O] | 3.54 | B:TRP 83[CD2] |  |  |  |
| A:CYS192[O] | 3.03 | B:ARG 100[CZ] | A:CYS 194[O] | 3.73 | B:TRP 83[CE3] |  |  |  |
| A:CYS192[O] | 3.01 | B:ARG 100[NH1] | A:CYS 194[O] | 3.55 | B:TRP 83[CZ2] |  |  |  |
| A:CYS192[O] | 3.47 | B:ARG 100[NH2] | A:CYS 194[O] | 3.70 | B:TRP 83[CZ3] |  |  |  |
| A:CYS192[CB] | 3.68 | B:TRP 82[CZ3] | A:CYS 194[O] | 3.61 | B:TRP 83[CH2] |  |  |  |
| A:CYS192[CB] | 3.74 | B:ARG 100[CB] | A:CYS 194[O] | 3.81 | B:ILE 102[CD1] |  |  |  |
| A:CYS192[SG] | 3.56 | B:ARG 100[N] | A:CYS 194[CB] | 3.74 | B:TRP 83[CZ3] |  |  |  |
| A:SER 193[N] | 3.68 | B:TRP 82[CG] | A:CYS 194[CB] | 3.88 | B:ILE 102[N] |  |  |  |
| A:SER 193[N] | 3.76 | B:TRP 82[CD2] | A:CYS 194[CB] | 3.86 | B:ILE 102[CB] |  |  |  |
| A:SER 193[CA] | 3.56 | B:TRP 82[CD1] | A:CYS 194[SG] | 3.84 | B:SER 101[CA] |  |  |  |
| A:SER 193[CA] | 3.79 | B:TRP 82[NE1] | A:CYS 194[SG] | 3.83 | B:SER 101[CB] |  |  |  |
| A:SER 193[C] | 3.89 | B:TRP 82[CG] | A:CYS 194[SG] | 3.51 | B:ILE 102[N] |  |  |  |
| A:SER 193[C] | 3.52 | B:TRP 82[CD1] | A:SER 195[N] | 3.66 | B:TRP 83[CG] |  |  |  |
| A:SER 193[O] | 3.34 | B:TRP 82[CA] | A:SER 195[N] | 3.70 | B:TRP 83[CD2] |  |  |  |
| A:SER 193[O] | 3.69 | B:TRP 82[C] | A:SER 195[CA] | 3.73 | B:TRP 83[CG] |  |  |  |
| A:SER 193[O] | 3.65 | B:TRP 82[CB] | A:SER 195[CA] | 3.54 | B:TRP 83[CD1] |  |  |  |
| A:SER 193[O] | 3.81 | B:TRP 82[CG] | A:SER 195[CA] | 3.70 | B:TRP 83[NE1] |  |  |  |
| A:SER 193[O] | 3.78 | B:TRP 82[CD1] | A:SER 195[C] | 3.79 | B:TRP 83[CA] |  |  |  |
| A:SER 193[O] | 3.12 | B:ILE 83[N] | A:SER 195[C] | 3.71 | B:TRP 83[CG] |  |  |  |
| A:SER 193[O] | 3.44 | B:ILE 83[CG1] | A:SER 195[C] | 3.43 | B:TRP 83[CD1] |  |  |  |
| A:SER 194[N] | 3.90 | B:TRP 82[CD1] | A:SER 195[O] | 3.25 | B:TRP 83[CA] |  |  |  |
| A:SER 194[CA] | 3.84 | B:ASP 81[O] | A:SER 195[O] | 3.49 | B:TRP 83[C] |  |  |  |
| A:SER 194[CB] | 3.78 | B:ASP 81[O] | A:SER 195[O] | 5.56 | B:TRP 83[CB] |  |  |  |
| A:ILE 196[CG1] | 3.78 | B:ILE 83[CG1] | A:SER 195[O] | 3.78 | B:TRP 83[CG] |  |  |  |
|  |  |  | A:SER 195[O] | 3.86 | B:TRP 83[CD1] |  |  |  |
|  |  |  | A:SER 195[O] | 2.82 | B:ILE 84[N] |  |  |  |
|  |  |  | A:SER 195[O] | 3.85 | B:ILE 84[CA] |  |  |  |
|  |  |  | A:SER 195[O] | 3.81 | B:ILE 84[CB] |  |  |  |
|  |  |  | A:SER 195[O] | 3.35 | B:ILE 84[CG1] |  |  |  |
|  |  |  | A:SER 195[O] | 3.80 | B:ILE 84[CD1] |  |  |  |
|  |  |  | A:GLY 196[N] | 3.63 | B:TRP 83[CD1] |  |  |  |
|  |  |  | A:GLY 196[CA] | 3.53 | B:ASP 82[O] |  |  |  |
|  |  |  | A:ASN 197[C] | 3.86 | B:ILE 84[CD1] |  |  |  |
|  |  |  | A:ASN 197[O] | 3.77 | B:ILE 84[CD1] |  |  |  |
|  |  |  | A:ILE 198[CG1] | 3.75 | B:ILE 84[CG1] |  |  |  |
|  |  |  | A:LEU 201[CD2] | 3.89 | B:ILE 84[CG1] |  |  |  |


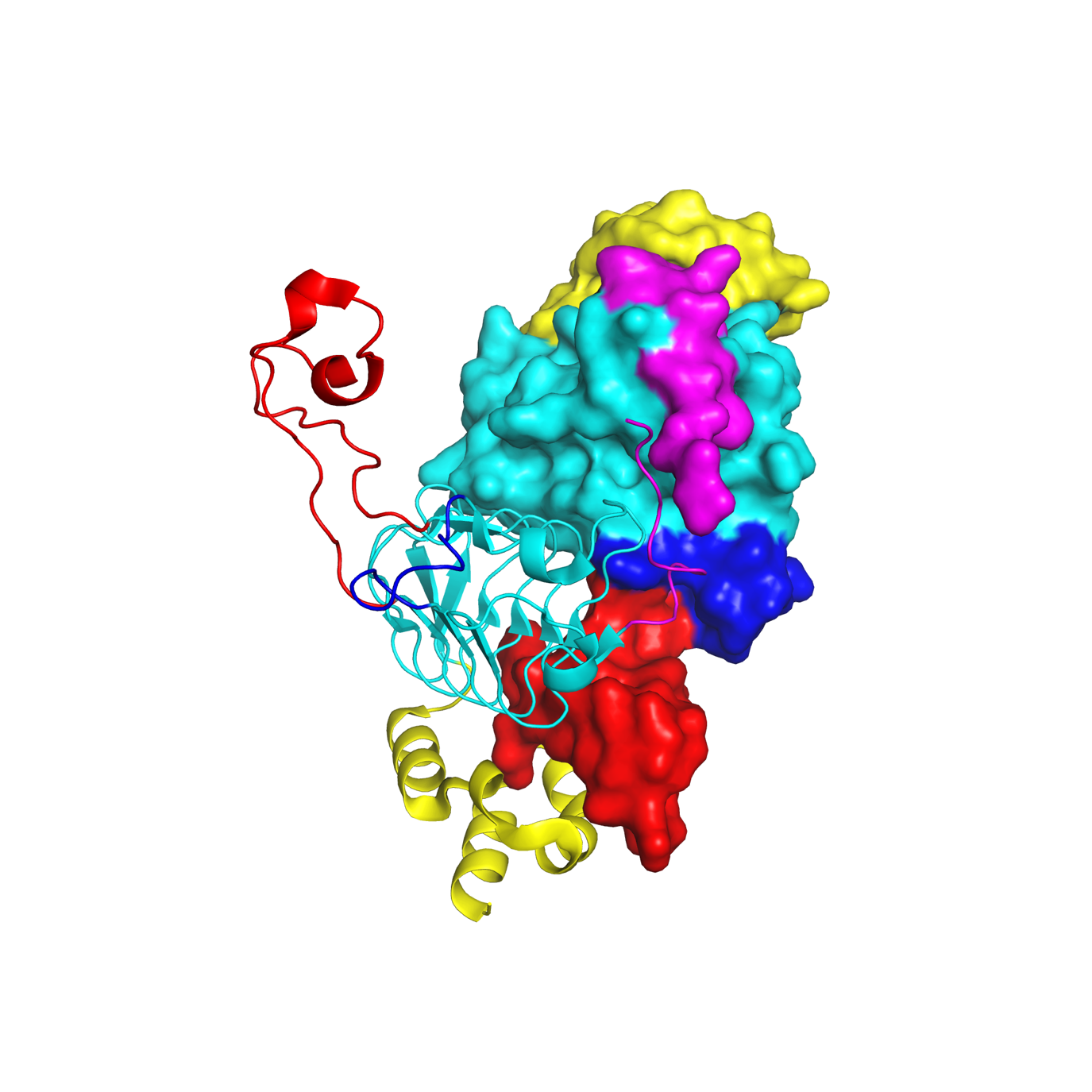


**Supplementary Figure 1**: **Potential structure of A/B interface of *E. anophelis* CAT B.** Main domains have been colored the same as in Figure 2.


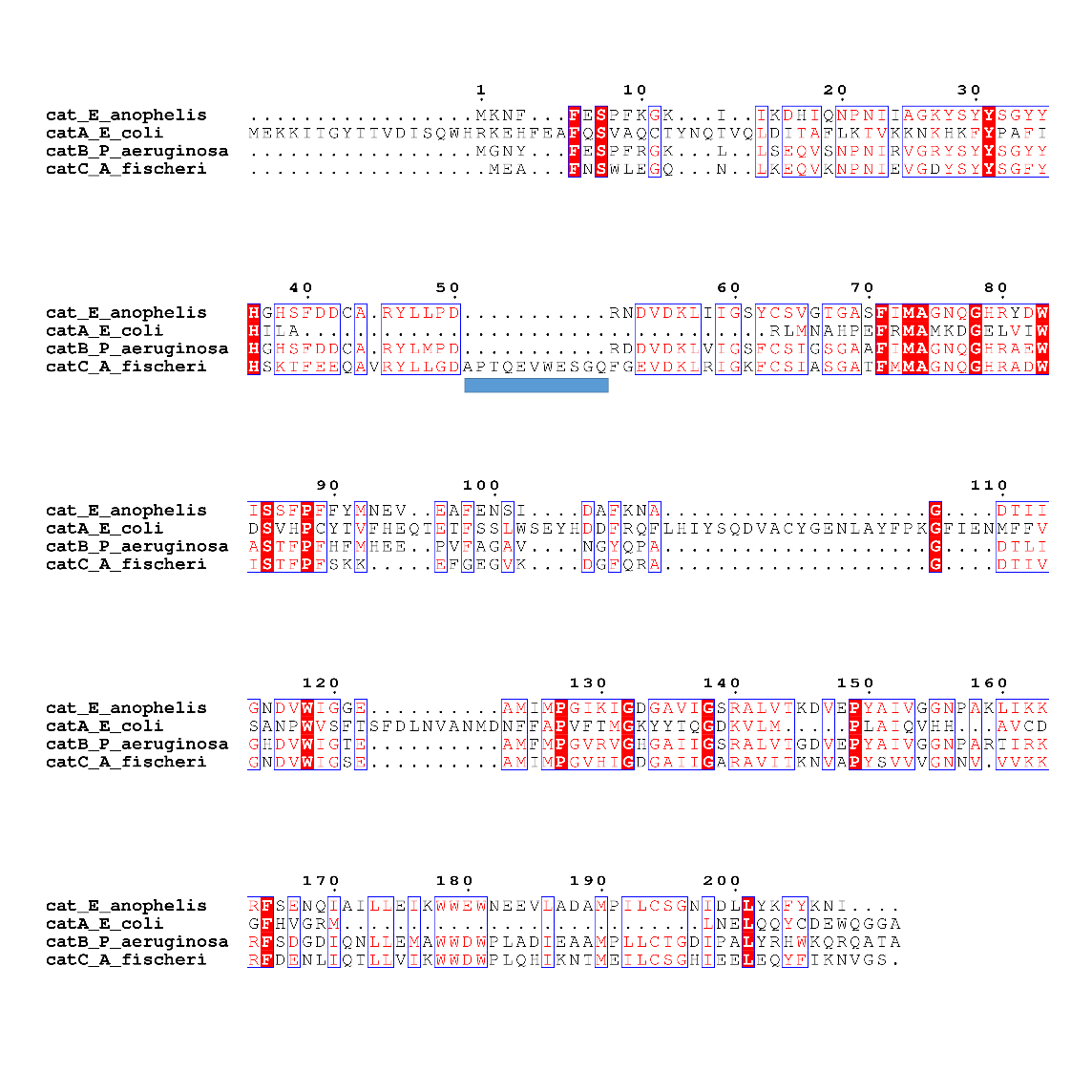


**Supplementary Figure 2. Multiple sequence alignment comparing selected Type A, B, and C CATs with *E. anophelis NUHP1* CAT protein.** Conserved residues are highlighted in red. The blue rectangle indicated the insertion sequence found in Type C CATs, but not Type B CATs. Protein sequences in the alignment included *Elizabethkingia anophelis* catB (UniProt ID A0A077EJ45), the Type A CAT from *Escherichia coli* catI (UniProt ID P62577), Type B CAT from *Pseudomonas aeruginosa* catB7 (UniProt ID P26841), and Type C CAT from *Allivibrio fischeri* catC (UniProt ID Q5DZD6). The multiple sequence alignment was generated with ESpript (http://espript.ibcp.fr/ESPript/ESPript/).
